# Interpersonal Physiological Synchrony During Dyadic Joint Action Is Increased by Task Novelty and Reduced by Social Anxiety

**DOI:** 10.1111/psyp.70031

**Published:** 2025-03-17

**Authors:** Sarah Boukarras, Valerio Placidi, Federico Rossano, Vanessa Era, Salvatore Maria Aglioti, Matteo Candidi

**Affiliations:** ^1^ Department of Psychology Sapienza University of Rome Rome Italy; ^2^ Santa Lucia Foundation (IRCCS) Rome Italy; ^3^ School of Advanced Studies, Centre for Neuroscience University of Camerino Camerino Italy; ^4^ Italian Institute of Technology, Sapienza University of Rome and CLN^2^S@Sapienza Rome Italy

## Abstract

Interpersonal physiological synchrony refers to the temporal coordination of autonomic states during social encounters. Previous studies indicate that physiological synchrony may arise during nonverbal interactions. Nevertheless, the role played by contextual and individual factors in determining its emergence is understudied. In this work, we examined heart rate synchrony during a cooperative joint action task, exploring how task constraints, novelty, and behavioral synchrony influence physiological alignment. To achieve this, we periodically modulated task demands by alternating between peer‐to‐peer and leader–follower dynamics, as well as between complementary and imitative movements, and their combinations. Additionally, we assessed the role of individual differences by examining the impact of dyad members' Social Anxiety and Perspective Taking levels. We further investigated how task demands and personal traits shape the perceived quality of social interactions and subject‐level heart rate variability. Our findings revealed a significant increase in physiological synchrony and a decrease in perceived interaction quality when participants switched to a novel task version (i.e., during switch blocks) compared to task repetition. Task switching was also associated with increased heart rate variability. Notably, Social Anxiety negatively predicted physiological synchrony, suggesting that more socially anxious dyads were less likely to achieve physiological alignment. However, no relationship was observed between physiological synchrony and task performance. Overall, our results suggest that physiological synchrony intensifies when dyads navigate the challenge of learning a novel task together, and that both contextual and individual aspects contribute to its emergence.

## Impact statement

This study examines how contextual and individual factors modulate the emergence of autonomic synchrony during nonverbal interactions. Results show that cardiac synchrony increases when the dyad needs to learn a new version of the joint action task. Moreover, dyads with higher levels of Social Anxiety display a reduced amount of synchrony. The findings hint to a plausible role of interpersonal monitoring and reciprocal attention processes in determining the emergence of interpersonal physiological synchrony.

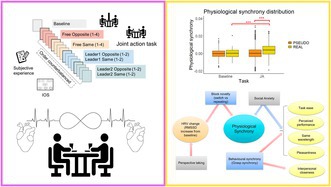

## 1 Introduction

During social interactions, people tend to synchronize their behavioral, neural, and autonomic states. In particular, physiological synchrony—the temporal alignment of autonomic activity—has been observed across a variety of social encounters and multiple physiological signals (Palumbo et al. 2017). Mounting evidence indicates that dyads and groups synchronize their cardiac, respiratory, and electrodermal activity (EDA) when discussing personal topics (Asher et al. 2021; Coutinho et al. 2021), drumming (Gordon, Gilboa, Cohen and Kleinfield, 2020; Gordon et al. 2020) and singing together (Müller and Lindenberger 2011), during psychotherapy (Messina et al. 2013; Palmieri et al. 2018; Tschacher and Meier 2020), cooperative problem‐solving (Behrens et al. 2020; Danyluck and Page‐Gould 2019), speed dates (Prochazkova et al. 2021; Zeevi et al. 2022), and mother–child interactions (Feldman et al. 2011; Feldman 2012).

While previous studies have primarily focused on conversations or cooperative tasks involving both verbal and nonverbal communication (Asher et al. 2021; Coutinho et al. 2021; Prochazkova et al. 2021; Danyluck and Page‐Gould 2019), the emergence of physiological synchrony in purely nonverbal tasks remains less explored (e.g., Noy et al. 2015; Gordon et al. 2020, 2023; Flory et al. 2023). In nonverbal joint action tasks, two or more people need to coordinate each other's movements in space and time in order to achieve a shared goal (Sebanz et al. 2006). This ability requires processes of reciprocal prediction and monitoring, perception–action integration and perspective taking (Sacheli et al. 2022) occurring simultaneously in the dyad or group members. This alignment of sensorimotor, cognitive, and attentional states may result in the emergence of autonomic synchrony, as previously observed during group drumming (Gordon et al. 2020) and during the Mirror Game, a naturalistic joint action task where two participants mimic each other's movements, creating coordinated, dance‐like sequences in unison (Noy et al. 2015). However, the role played by contextual and individual factors in determining or modulating the emergence of autonomic synchrony in joint actions is understudied. Moreover, the relationship between physiological synchrony and behavioral alignment or—more in general—task performance has not been clearly defined (see, e.g., Mayo et al. 2021; Gordon et al. 2024) and requires further investigation.

In this exploratory study, we first aimed at investigating how cardiac synchrony emerging in joint action is modulated by a set of contextual demands that typically vary during motor interaction tasks (leader–follower roles, task novelty) and by dyad‐level differences in willingness and ability to interact (Social Anxiety and Perspective Taking). Cardiac activity, the length of inter‐beat intervals (IBIs), varies continuously to facilitate the organism's adaptation to the environment, for example, slowing down in response to salient stimuli and accelerating during action execution (Skora et al. 2022). Therefore, cardiac synchrony is particularly suitable to study the physiological bases of reciprocal monitoring and adaptation during joint action. Additionally, we explored whether heart rate variability (HRV)—the length variation of consecutive IBIs regulated by the parasympathetic nervous system through the vagus nerve—was modulated by task demands and whether it could predict the emergence of cardiac synchrony. Finally, we explored the interplay between behavioral and autonomic synchrony and the subjective feelings elicited by the interaction.

In the following paragraphs, we specify the research questions and hypotheses driving the current work.

### 1.1 RQ1: Which Factors Promote the Emergence of Physiological Synchrony During Joint Action?

To answer this RQ, we investigated the impact of behavioral synchrony; task‐related contextual demands (peer‐to‐peer vs. leader–follower and task novelty); and individual differences in interpersonal disposition traits and cognitive skills, that is, Social Anxiety, Perspective Taking, and subject‐level HRV, on physiological synchrony.

Concerning the interplay between behavioral and autonomic synchrony, previous studies have indeed reported a positive relationship (Gordon et al. 2020; Noy et al. 2015). Nevertheless, the direction of this relationship is unclear. Specifically, while cardiac synchrony may drive the alignment of the interactors' sensorimotor states, it is equally plausible that the sensorimotor alignment itself gives rise to patterns of cardiac synchrony. Moreover, in a recent study using wavelet coherence to quantify cardiac synchrony in dyads engaged in a social finger‐tapping task, although task‐related synchrony increased compared to a resting baseline, it was not different from random synchrony (Flory et al. 2023). Thus, the interplay between different synchrony modalities may depend on the type of task at play and on the interaction context (see Gordon et al. 2024).

Autonomic synchrony during joint action seems to be modulated by task‐related constraints, such as whether the task is presented as competitive or cooperative (Chanel et al. 2012; Danyluck and Page‐Gould 2019; Järvelä et al. 2014). Leader–follower dynamics, while extensively studied in verbal interactions (e.g., Kraus and Mendes 2014; Vigier et al. 2021; Thorson et al. 2021; Guastello et al. 2020), still need to be explicitly examined in the context of joint actions. Leader–follower nonverbal tasks are characterized by an asymmetric distribution of information between co‐actors, where the leader has access to task instructions, while the follower must rely on the leader's actions to achieve the shared goal (Konvalinka et al. 2014; Candidi et al. 2015; Dumas and Fairhurst 2021; Gallotti et al. 2017). This reduction in mutual exchange of information may dampen physiological synchrony. Additionally, leader–follower dynamics involve a temporal delay, with the leader initiating the movement and the follower responding accordingly. Research suggests that in asymmetric interactions, physiological synchrony is defined by temporal patterns of directional influence (Thorson et al. 2018), with synchrony emerging only at specific time lags. This results in the leader's physiological states predicting subsequent changes in the follower's states (Boukarras et al. 2024). Based on these findings, we hypothesize that physiological synchrony in leader–follower conditions, compared to “free” interaction blocks, will be either diminished or observable only at later time lags.

Leveraging our experimental design, which includes multiple task conditions, we investigated how task novelty influences the emergence of physiological synchrony. Specifically, we compared levels of synchrony measured during task‐switching blocks, where the dyad had to learn a new task condition, with those in repetition blocks. At the (individual) physiological level, stimulus novelty and task complexity are known to elicit transient changes in heart rate, consistent with attentional reorientation processes (see Skora et al. 2022, for review). While no study to date has explicitly examined how physiological synchrony is affected by task novelty, two competing hypotheses can be proposed. On one hand, it is possible that as the dyad learns the task rules and becomes more skilled, their physiological states become increasingly aligned. This alignment could be disrupted when the task rules change. On the other hand, repeating the same task might lead to boredom. Evidence from a recent study using the Mirror Game (Ravreby et al. 2022) suggests that people prefer engaging in novel movements over repeated ones, even at the expense of behavioral synchrony. Boredom, in turn, may shift attention away from the social context, prompting mind wandering or unrelated thoughts and reducing the need for interpersonal performance monitoring, which is key to coordinating in dynamic interactions (Moreau et al. 2023). In such cases, while the dyad may continue synchronizing their movements, their cognitive and attentional processes may become misaligned. Introducing a change in task rules could re‐engage participants, prompting a return to a “social monitoring” state and realigning their physiological activity.

While context may play a crucial role in providing a foundation for interpersonal synchrony, the contributions of each individual to the interaction should not be overlooked. In particular, individual differences—especially in the ability and willingness to engage in social interaction—can significantly influence the likelihood of synchrony emerging. Previous research provided initial evidence that Social Anxiety may disrupt physiological synchrony. Asher and colleagues (2021) observed a negative relationship between Social Anxiety levels and physiological synchrony during closeness‐generating conversation only in dyads including a diagnosed socially anxious individual. Similarly, in a group‐drumming task, cardiac synchrony was negatively related to group‐level (but not to individual‐level) Social Anxiety (Gordon et al. 2023). In addition, a recent study showed that people high in avoidant attachment are more likely to display synchronization patterns compatible with social disengagement (Boyd et al. 2022).

Conversely, multiple studies have reported a positive effect of empathic traits on physiological synchrony (Chatel‐Goldman et al. 2014; Järvelä et al. 2014), while parasympathetic cardiac linkage between a person reporting a stressful event and a listener was found to be associated with the listeners' empathic accuracy (Brown et al. 2021). Although causality remains to be established, these findings suggest that the superior ability of highly empathetic individuals to perceive others' emotions may be closely linked to their stronger tendency to get “in sync” with others (West and Mendes 2023). One empathy facet that might be more relevant for joint action research is Perspective Taking, the ability to adopt and understand the point of view of another person. Previous studies found that this cognitive skill improves motor coordination in joint action, possibly by promoting accuracy in predicting each other's behavior and sensitivity to other‐related information (Novembre et al. 2012, 2019; Sacheli et al. 2022). While the role of Perspective Taking in physiological synchrony still needs to be understood, it is conceivable that people with this skill will more easily align their autonomic states when asked to predict and coordinate each other's actions. Thus, we hypothesize that dyads higher in Perspective Taking will display more physiological synchrony.

Another important aspect that should be taken into account is the dyad members' physiological state during the interaction. On one hand, high levels of HRV are associated with better interaction quality (Shahrestani et al. 2015), which may enhance interpersonal physiological synchrony. On the other hand, HRV can be understood as a time series produced by a nonlinear oscillator. When the variability in the time series is lower, the stability and predictability of the signal increase. In the context of physiological synchrony, individuals with lower HRV levels may align more easily with each other due to the greater predictability of their respective signals. We, therefore, hypothesize that HRV will be either positively or negatively related to physiological synchronization. In addition, HRV itself might be modulated by task demands. Thus, we formulated a second research question:

### 1.2 RQ2: Are Task‐Related Changes in HRV Modulated by Contextual and Individual Factors?

Previous studies have mainly focused on HRV measured at rest as a predictor of cognitive functions and mental health (Thayer et al. 2009; Forte et al. 2019). Conversely, task‐induced changes in cardiac variability are less studied. Previous research converges in demonstrating that social stressors (Allen and Friedman 2016), worry and rumination (Ottaviani et al. 2016; Era et al. 2021), and cognitive tasks requiring increased attention (see, e.g., Hansen et al. 2003; Balzarotti et al. 2017 for review) lead to a phasic reduction in vagally mediated HRV (i.e., vagal withdrawal). A recent publication (Smith et al. 2020) instead reviewed research concerning task‐evoked increases in parasympathetic HRV, which were observed in response to stimuli‐inducing compassion, during emotional regulation, and during behavioral self‐regulation. Interestingly, some of the reviewed articles also report a positive relationship between HRV increases and engagement (Williams et al. 2009) or attentiveness (Cribbet et al. 2011) during social interactions. Overall, the sparse literature suggests a connection between increases in HRV and enhanced self‐regulation and engagement—both of which may support effective reciprocal monitoring in joint actions. However, task‐related changes in HRV could also be influenced by individual predispositions toward social interaction. For instance, individuals with high levels of Social Anxiety might perceive social interactions as more stressful, leading to a decrease in HRV rather than an increase. This brings us to the third and final research question:

### 1.3 RQ3: What Factors Influence the Subjective Experience of the Interaction?

Previous research indicates that behavioral synchrony, both spontaneous and instructed, is perceived as a rewarding and pleasurable experience, in some cases leading to positive outcomes such as prosociality and interpersonal liking (see Mogan et al. 2017). However, whether some interaction modalities are experienced as more pleasant than others is unclear. For example, while blocks of interactions performed under novel rules should be perceived as more pleasant (see Ravreby et al. 2022), do people enjoy coordinating with others more when there are designed leader–follower roles compared to “free” interactions? Another important question is whether physiological alignment is associated with pleasant or otherwise subjective experiences. While in some studies physiological synchrony was found to be related to positive affect (Chanel et al. 2012; Danyluck and Page‐Gould 2018), empathy (Chatel‐Goldman et al. 2014), and group cohesion (Gordon et al. 2020), others observed a positive relationship between physiological synchrony and *negative* affect (Wilson et al. 2018) or distress in couples (Levenson and Gottman 1983), suggesting that physiological synchrony does not necessarily reflect a positive evaluation of the interaction. Therefore, in the present study, we sampled participants' subjective feelings elicited by the interaction and investigated which contextual, individual, and physiological predictors were associated with perceived alignment, performance, task ease, and pleasantness.

## 2 Methods

### 2.1 Participants

Forty‐five same‐sex unacquainted dyads were recruited from the CoSAN Lab (Sapienza University) database or through leaflets and social media posts. We chose to focus on same‐sex, unacquainted dyads to control for potential confounding variables that could influence physiological synchrony. Previous research suggests that factors such as gender composition (e.g., mixed‐sex vs. same‐sex dyads) and preexisting relationships between partners can affect interaction dynamics (Bizzego et al. 2019). Exclusion criteria were any diagnosis of psychiatric, neuropsychological, or cardiac conditions and left‐handedness. From the initial sample, two dyads were excluded from the study due to malfunctioning of the joint action task setup, while three other dyads were excluded because their physiological recordings were too noisy. The final sample included 80 participants aged 18–40 (*M* = 25.8, SD = 4.19) paired in 40 dyads (17 MM and 23 FF). The sample size was based on previous studies using a similar experimental design and physiological synchrony measure (e.g., Gordon, Gilboa, Cohen and Kleinfield, 2020; Gordon et al. 2020; Noy et al. 2015; Henning and Korbelak 2005; Järvelä et al. 2014). Based on two recently published meta‐analyses examining the relationship between physiological synchrony, interaction perception, and behavioral synchrony (Mayo et al. 2021; Ohayon and Gordon 2024), we expected to observe small effect sizes, which would require larger samples. However, given that data collection started in 2021, during a time when Italy experienced frequent lockdowns due to the COVID‐19 pandemic, we anticipated that recruiting and testing more than 80 participants would present significant challenges.

### 2.2 General Procedure

**FIGURE 1:**
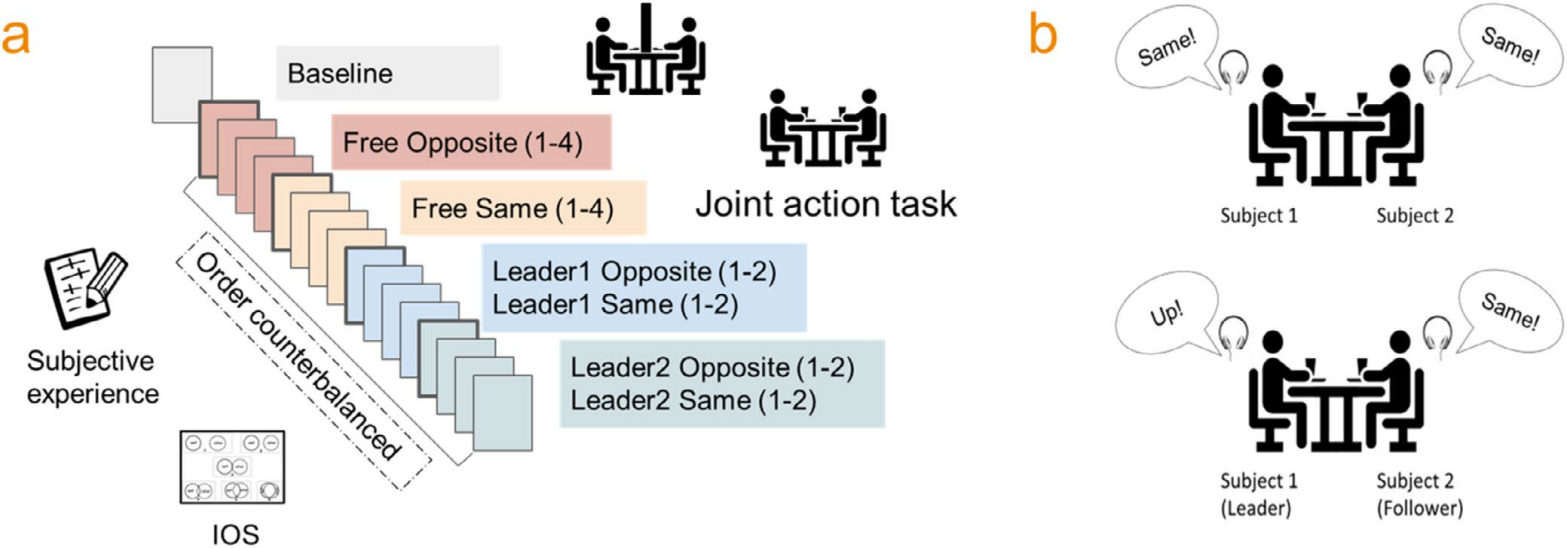
Panel (a)—Experiment design. The experiment started with a baseline HR recording in which participants were seated at the table and were asked to relax while a polystyrene panel was placed in between to prevent them from seeing each other. After the baseline and a trial block, participants completed 16 blocks of the joint grasping task in four different conditions (order counterbalanced). At the end of each block, they were asked to fill out the Subjective Experience questionnaire, while at the end of each of the four conditions, they completed the Inclusion of Other in the Self (IOS, Aron et al. 1992) questionnaire. Panel (b)—Joint grasping task. In the free blocks (upper image), both participants received the same instruction, while in the leader–follower blocks (lower image), the leader received instructions regarding the grasping to be performed (precision/up or power/down), while the follower was asked to perform either an imitative (same) or a complementary (opposite) movement.

Participants were asked to refrain from caffeine and nicotine for at least 1 h before the start of the experiment to minimize potential confounding effects on heart rate. Both caffeine and nicotine are known stimulants that can influence cardiovascular activity, specifically increasing the heart rate. Upon their arrival at the laboratory, participants were randomly assigned either to the Subject 1 or the Subject 2 side of the joint action table and received a detailed explanation of the experiment. Participants were then attached to the ECG electrodes, the copper plates for the joint action task, and the reflective markers for kinematics. Before starting the baseline ECG recording, a polystyrene panel was placed on the table so that the two participants could not see each other. At the end of the baseline recording (lasting approximately 4 min), participants completed 16 blocks of the joint grasping task (20 trials per block), while their cardiac activity was recorded. The blocks (each lasting approximately 5 min) were interspersed with pen‐and‐pencil Subjective Experience questionnaires (see Figure 1a). The total duration of the experiment was around 2 h. Before leaving the laboratory, participants were fully debriefed about the experimental hypotheses and allowed to ask questions. The experimental procedure was approved by the Santa Lucia Foundation (Rome)'s ethics committee. Data were collected between November 2021 and March 2023. All participants wore surgical or FFP2 face masks during all the experimental phases, in compliance with the local COVID‐19 safety measures.

### 2.3 Joint Grasping Task

The joint grasping task (Boukarras et al. 2021; Candidi et al. 2015; Era et al. 2018, 2020, 2023; Moreau et al. 2020; Sacheli et al. 2012) taps into multiple motor and cognitive aspects of joint action, including behavioral synchrony, action prediction, reciprocal monitoring, and mutual adjustment. The task requires two facing participants to grasp two bottle‐shaped objects as synchronously as possible while following auditory instructions specifying different coordination modes. Task performance is operationalized as the interpersonal synchrony in grasping the objects (grasp time difference). However, we also recorded participants' synchrony in initiating the grasping movements (Start Time Difference) as a measure of spontaneous behavioral synchrony.

We used two versions of the task, one (Free task) in which both participants received the same instruction, namely, to perform either an imitative (Same) or complementary (Opposite) movement with respect to the other participant, and another (Leader–Follower task) where one member (the Leader) received instructions regarding the action to be performed (i.e., grasp the upper or the lower part of the bottle), while the other (the Follower) was asked to perform a complementary or imitative movement. Opposite and same blocks were mainly introduced to create variability in the task, which otherwise would become too repetitive. While we expected no differences between the two conditions, both requiring similar processes of action monitoring and prediction (see Sacheli et al. 2018), in terms of behavioral and physiological synchrony, we were interested in sampling the subjective feelings associated with performing imitative versus complementary actions.

Participants were seated at the opposite sides of a wooden table (120 × 100 cm) facing each other, and were instructed to reach and grasp one of two bottle‐shaped objects placed in front of them (40 cm away from the participant and 5 cm from the midline) following auditory instructions delivered via headphones (see Figure 1b). The bottle‐shaped objects were constituted of two superimposed cylinders with different diameters (small, 2.5; large, 7.0 cm). Given their shape, the upper cylinder was to be grasped with a precision grip (index finger and thumb), while the lower cylinder was to be grasped with a power grip (whole hand). On each cylinder, two pairs of touch‐sensitive copper plates were placed at 15 and 23 cm of the total height of the object. Two other pairs of copper plates were placed on the thumb and index finger of participants. Before the start of each trial, participants were asked to keep pressing between their right index and thumb fingers a starting button placed 40 cm away from the bottle‐shaped object and 10 cm to the right of the midline. Start movement time was recorded from button release. After instruction delivery, participants were instructed to grasp the bottle‐shaped object while ensuring that the copper plates placed on their fingers were aligned with the plates on the bottle (both in the precision and in the power grip). This copper‐to‐copper contact triggered an electric signal that was recorded and stored as participants' touch times. Instruction delivery and response collection were managed through E‐Prime 2 Pro (Psychology Software Tools Inc., Pittsburgh, PA). Auditory instructions concerning the movement to be executed were delivered simultaneously to both participants via headphones. The instructions took the form of a human voice uttering one of the following words: “same,” “opposite,” “high,” or “low,” depending on the experimental task (see below). Participants were asked to wait until the end of the auditory instruction before starting the reach‐to‐grasp movement. In the Free task, both participants received identical instruction, which could be either the word “opposite” or the word “same,” varying by block. During “opposite” blocks, participants had to grasp the bottle‐shaped objects in a complementary fashion (i.e., one of the two had to grasp the upper cylinder with a precision grip, while the other had to grasp the lower one with a power grip), while “same” blocks required participants to grasp their bottles in the same location. No explicit instructions were given regarding how they should reach an agreement about where to grasp the bottles, but participants were not allowed to talk and were discouraged from repeating the same combination (e.g., both grasping the lower cylinder) more than once. The free task was repeated for eight blocks (four “opposite” and four “same”), each comprising 20 trials. In the leader–follower task, one of the two members was assigned the role of leader and received the instruction of grasping either the upper cylinder (instruction “high”) or the lower one (instruction “low”), varying by trial. The other participant (i.e., the follower) received either the “same” or the “opposite” instruction, depending on the block. Thus, the follower had to observe where the leader was going to grasp the bottle and then perform either an imitative or a complementary grasp. After four blocks (two “opposite” and two “same”), participants switched their roles so that the leader became the follower and the follower became the leader and completed four more blocks. The leader–follower task thus comprised eight blocks, of which four were categorized as “Leader 1” (i.e., Subject 1 was the leader) and four as “Leader 2”. Movement kinematics were tracked and recorded with a SMART‐D motion capture system (Bioengineering Technology & Systems [B|T|S]). Each participant had three infrared reflective markers (5 mm diameter), each attached to (i) thumb, ulnar side of the nail; (ii) index finger, radial side of the nail; and (iii) wrist, dorso‐distal aspect of the radial styloid process. Four infrared cameras with wide‐angle lenses (sampling rate 100 Hz) were placed about 100 cm away from each of the four corners of the table to capture the movements of the markers in the 3D space. Kinematic data were collected but will not be reported in this article.

### 2.4 Subjective Experience Sampling

Participants' subjective experiences of the interaction were collected at the end of each block, by asking them to rate their level of agreement with the following statements: “We were good at touching the bottles at the same time” (Performance goodness), “Coordinating with my partner was easy” (Easiness), “Coordinating with my partner was pleasant” (Pleasantness), and “I felt like my partner and I were on the same wavelength” (Same wavelength) using paper‐and‐pencil visual analog scales (VAS) ranging from 0 (*not at all*) to 100 (*very much*). In addition, participants were asked to rate who they thought contributed more to the success of the interaction using a VAS ranging from 0 (*only me*) to 100 (*only my partner*). VAS scales, originally developed to measure pain levels in patients (Scott and Huskisson 1976), offer distinct advantages over traditional Likert scales. By allowing participants to rate their subjective experiences on a continuous scale with more points, VAS scales enhance sensitivity, enabling the detection of subtle changes that might be overlooked when using a more limited 7‐point scale. Every four blocks, namely, before switching to a different condition, participants completed the Inclusion of the Other in the Self scale (Aron et al. 1992) to assess their experienced level of interpersonal closeness with the interaction partner.

### 2.5 Individual Differences Questionnaires

We measured social anxiety and perspective taking using the Italian version of the Liebowitz Social Anxiety Scale (Baroni et al. 2019) and the Perspective Taking subscale of the Interpersonal Reactivity Index (Albiero et al. 2006) questionnaire.

### 2.6 Physiological Recording

ECG activity was continuously recorded from both participants during each experimental block through disposable Ag/Agcl electrodes placed on their left wrist and right and left ankles. The ECG signal was sent to a g.HIAMP biosignal amplifier (g.tec medical engineering GmbH—Austria). ECG data were acquired at a sampling frequency of 512 Hz and monitored online with the g.RECORDER software (g.tec medical engineering GmbH—Austria) and then saved in a digital format. Raw data were then processed in MATLAB (2020) with a customized semiautomatic algorithm (available at OSF_PhysioSynch) to apply a high‐pass filter with a 0.6 Hz cutoff frequency to remove baseline wandering and a low‐pass filter with a 20 Hz cutoff to remove the highest frequency components. From the filtered ECG data, we extracted the R‐peaks using the *findpeaks* MATLAB function. For each data file, the plotted peaks were visually inspected to check that the algorithm correctly identified each R‐peak. Wrongly identified peaks were manually deleted and replaced with the correct ones. IBIs time series were then computed as the difference in milliseconds between two consecutive R‐peaks. Outlier IBIs (approximately 2%–3% for each block) were eliminated via manual thresholding, and their values were replaced using a spline interpolation. Finally, the IBI series were resampled at 4 Hz to generate the data for the cross‐correlation analysis.

### 2.7 Cross‐Correlation of IBI Data

We estimated the presence of physiological synchrony in each experimental block using cross‐correlation analysis. Cross‐correlation applied to nonstationary and autocorrelated time series, such as cardiac signals, may lead to spurious results (Dean and Dunsmuir 2016). It is thus necessary to remove or at least minimize such components in order to obtain reliable estimates of correlation. As a first step, to remove autocorrelation, nonstationarity, and seasonal components (periodic trends superimposed to the signal) from the IBI series, each individual time series was submitted to an ARIMA(p,d,q) model (as in Feldman et al. 2011). ARIMA models are a generalization of ARMA(*p,q*) models and represent a form of regression analysis fitted to time‐series data. The ARIMA model is formed by an autoregression (AR) polynomial, an integrated (I) polynomial, and a moving average (MA) polynomial. The model's goodness of fit depends on the choice of three parameters: *p* the number of autoregressive parameters, *d* the order of differencing (i.e., representing the differencing step(s) of raw data to eliminate the nonstationary components), and *q* the number of MA terms. For each IBI series, we identified the best model parameters to fit our data using the auto‐ARIMA procedure implemented through the *auto.arima* R function (*forecast* package, Hyndman et al. 2023). We ran the model with *d* = 1, to perform the first‐differencing operation once, and seasonality order *D* = 1, to perform a first‐order seasonal differencing. In this way, nonstationary (time‐series mean ≠ 0) and seasonal components (trend, cyclical, irregular variations) found in both the series were removed or minimized. Other parameters used in the *auto.arima* function were as follows:

- The goodness‐of‐fit criteria were set to {“aicc”, “aic”, “bic”} (Corrected Akaike Information Criterion, Akaike Information Criterion, and Bayesian Information Criterion, respectively); in this way, the algorithm tries the ARIMA models generated and finds the optimal one using these three criteria.
- The “approximation” and “stepwise” parameters were set to false, allowing a more precise search of the best ARIMA model to fit the data, at the expense of higher computational time.
- Lambda parameter was set to “auto,” meaning that the data underwent a Box–Cox transform with the best lambda that minimized the coefficient of variation of the time series under investigation.

**FIGURE 2:**
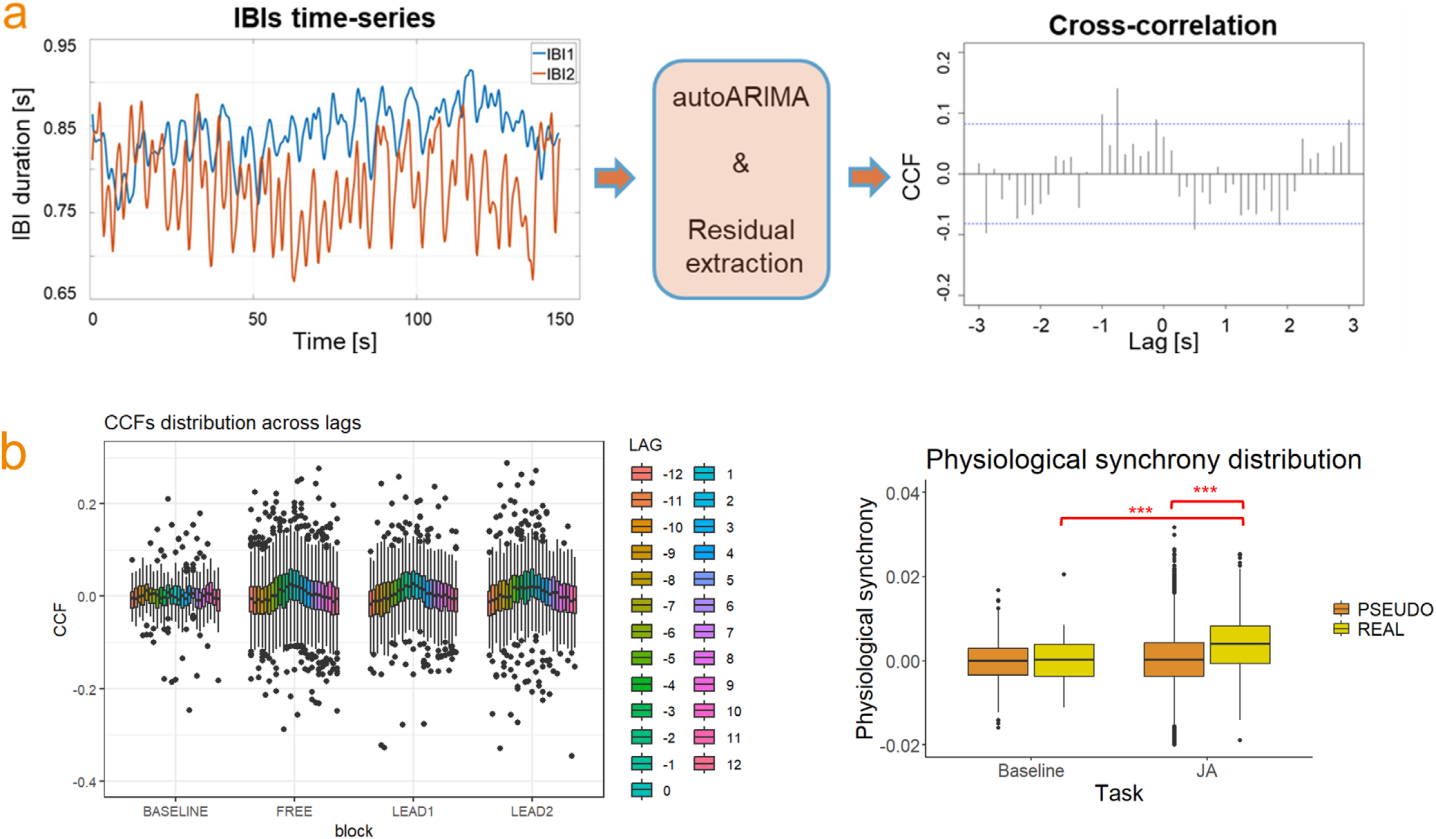
Panel (a) Computation of physiological synchrony. Individual IBI series during each block were submitted to the auto‐ARIMA procedure. Residuals from the best models were then extracted, and cross‐correlation between the two residual series was computed (cross‐correlation function [CCF]). Panel (b) Distribution of CCF values across the 25 lags (left plot) and distribution of physiological synchrony (averaged CCF values) in REAL versus PSEUDO dyads in joint action and baseline tasks (right plot).

After the best ARIMA model was found for each pair of IBI time series, we extracted the residuals. Such residuals will present removed or minimized non‐stationary and autocorrelated components compared to the original IBIs. Following this, we computed the cross‐correlation function (CCF) between the dyad's two series of residuals with a maximum time lag of ±3 s, which is equal to the trial duration. This lag time range equals ±12 lag samples because the IBI time series were sampled at 4 Hz. The total length of the computed CCF resulted in 25 samples for each block. We stored the CCF values (CCFs) at every time lag (see Figure 2a).

### 2.8 CCFs' Distribution Across Lags and Averaging

We further investigated how CCF values were distributed across lags in the various tasks. Our expectations were as follows. For the baseline task, we expected the CCF values to be equally and randomly distributed across lags. In the free task, we expected to find the highest CCF values around lags close to zero, indicating the presence of concurrent synchrony (compatible with a task in which both dyad members have the same role). In the leader–follower tasks, because leader–follower interactions should give rise to patterns of physiological synchrony that are characterized by longer temporal lags (i.e., it is expected that the leader's physiology predicts changes in the follower's physiology, see, e.g., Kraus and Mendes 2014), we expected the CCF values to be higher at either positive or negative lags—depending on whether the leader or the follower is taken as a reference in the CCF. We consistently performed cross‐correlation by sliding Participant 2's signal over Participant 1's reference signal for each condition and block. Because participants switched roles from Leader 1 to Leader 2 blocks, we expected to find higher CCF values at positive lags in Leader 1 and higher CCF values at negative lags for Leader 2 (indicating that the leader influenced the follower).

The distribution of CCFs in different experimental conditions across time lags can be seen in Figure 2b (left plot) and Figure S1 and indicates the presence of in‐phase (positive CCF) synchrony around lag zero for all joint action tasks. Importantly, CCF values computed from the pseudo dyads (i.e., from randomly paired IBI series; see the paragraph “Pseudo dyads” for a description of the method) were equally spread across lags (see Figure S2), revealing the absence of a clear pattern, as expected by randomly matched IBI series. As shown in Figure 2 and in Figure S1, CCF values (for real dyads) tended to increase around lag zero for all joint action tasks. This observation was corroborated analytically by dividing the CCF values into three groups according to the lag at which they occurred: group “Zero” (from lag −4 to lag 4), group “Negative” (from lag −12 to lag −3), and group “Positive” (from lag 5 to lag 12) (see Figure S4). We ran a linear mixed model to measure the influence of block (baseline, free, Leader 1, Leader 2), LagGroup (negative, zero, positive), and their interaction on the dependent variable CCF. The model includes a by‐dyad random intercept. The model yielded a significant Block × LagGroup interaction (*X* ^2^ = 41.75, *p* < 0.0001). As expected, in the baseline condition, there were no differences in CCF values for the three lag groups (all *P*s > 0.86). In the free task, CCF values were higher in the zero lag group compared to both positive (*p* < 0.0001) and negative (*p* < 0.0001) lag groups, which were not different from each other (*p* > 0.260). Contrary to our expectations, Leader 1 and Leader 2 blocks showed the same pattern, with higher CCFs in the zero lag group compared to negative and positive (all *P*s < 0.0001) and no significant negative–positive difference (Leader 1: *p* = 0.327; Leader 2: *p* = 0.622), see Figure S3.

As there was no evidence for lagged synchrony, we decided to average all the CCF values across all lags to obtain a single value of physiological synchrony per block/participant (physiological synchrony), following the procedure reported in previous studies (e.g., Coutinho et al. 2021). Control analyses focusing only on CCF values from the zero lag group are presented in the Supporting Information. However, different from other previous studies (e.g., Coutinho et al. 2021) and following the recommendation of Tschacher and Meier (2020), we kept the original (i.e., non‐absolute) CCF value to differentiate between in‐phase and antiphase synchrony. The physiological synchrony values were then *z*‐transformed using Fisher's *Z* transformation before being entered into regression models. Descriptive statistics for Average_CCF across task conditions are presented in Table 1.

**TABLE 1:** Dependent variables across task conditions.

	Block novelty		Block type		Movement	
	Old	New	Free	L‐F	Same	Opposite
Physiological synchrony	**0.35 (0.7)**	**0.55 (0.7)**	0.32 (0.70)	0.48 (0.71)	0.39 (0.73)	0.40 (0.69)
HRV change	**−0.26 (4.2)**	**0.31 (4.7)**	−0.22 (4.3)	−0.01 (4.4)	−0.13 (4.3)	−0.10 (4.3)
Performance goodness	**79.9 (15.8)**	**73.9 (18.6)**	77.2 (16.8)	79.7 (16.6)	79.9 (16.3)	**77 (17.1)**
Easiness	**78.5 (16.7)**	**71.8 (20.1)**	**75.4 (17.6)**	**78.3 (18)**	78.5 (17)	**75.2 (18.5)**
Pleasantness	**77.6 (19.2)**	**74.9 (19.3)**	77.3 (18.7)	76.6 (19.9)	77.7 (18.9)	**76.2 (19.7)**
Same wavelength	**77.6 (17.3)**	**72.9 (19.5)**	76.2 (17.7)	76.6 (18.2)	77.7 (17)	**75.1 (18.8)**
IOS			4.1 (1.1)	3.8 (1.3)	**4 (1.2)**	**3.91 (1.2)**

*Note:* Means and standard deviations of physiological synchrony, HRV change, and subjective ratings of the interaction experience. Significant differences in task conditions are highlighted in bold. “L‐F” indicates leader–follower blocks. Physiological synchrony values are multiplied by 100 to make them more readable.

### 2.9 Pseudo Dyads

Detecting physiological synchrony between the IBI series of two interacting individuals cannot exclude the possibility that the observed synchronization merely reflects the fact that both participants are engaged in the same task/condition. For this reason, it is important to compare the distribution of observed physiological synchrony values with a distribution of values coming from surrogate dyads performing the same task/condition (Altmann et al. 2022; Lübbert et al. 2024; Ramseyer 2019; Tschacher and Meier 2020; Behrens et al. 2020). To this end, we applied an approach known as “participant shuffling” (Moulder et al. 2018; Bernieri et al. 1988). We generated a pseudo dataset of *N* = 13.260 IBI time‐series pairs, each representing the cardiac activity of individuals who had never interacted with each other. Within each block and condition, we matched the IBI series of Subject 1 from one dyad with the IBI series of Subject 2 from a different dyad. We then applied the same auto‐ARIMA procedure described in the “Cross‐Correlation of IBI Data” paragraph to series coming from the same condition (i.e., same “Block Type”, same “Movement,” and same session number), but from participants who were not part of the same dyad (and thus never interacted). The number of random pairs (13.260) was determined with the following equation:

$$N_{p}=\frac{N_{b}^{2}-N_{b}}{2}\cdot N_{c}$$

where *N*_*p*_ is the number of random pairs needed to ensure that each block for each dyad is paired with all the other dyad–block combinations in the same condition, *N*_*b*_ is the number of blocks in the same condition across dyads (*N* = 40), and *N*_*c*_ is the number of conditions for each dyad (*N* = 17, 16 experimental blocks and 1 baseline). Following the auto‐ARIMA procedure, we averaged the 25 CCF values to generate another distribution of physiological synchrony values that were again transformed using the Fisher's *Z* procedure. We compared the distribution of physiological synchrony values in randomly paired (PSEUDO) dyads against the distribution of the real (REAL) dyads separately for the baseline and joint action condition using Welch's *t*‐tests for unequal samples.

### 2.10 Heart Rate Variability (HRV) Calculation

We estimated the root mean square of successive RR interval differences (rMSSD, an index of vagally mediated beat‐to‐beat variance in heart rate, Shaffer and Ginsberg 2024) from individual IBI series in each experimental block and during the resting baseline using the MATLAB toolbox “HRV_tool” (Vollmer 2019). We then calculated for each experimental block an index of HRV change from the resting baseline block (HRV change = HRV_block—HRV baseline). Descriptive statistics for HRV change across task conditions are presented in Table 1.

### 2.11 Behavioral Variables

For each trial of the joint grasping task, we calculated two measures of behavioral synchrony: (1) the absolute difference between the two participants' grasping times (grasp time difference) and (2) the absolute difference between their reaction times, defined as the time from the auditory instruction to the release of the start button (start time difference). These measures were initially computed as the time differences (in milliseconds) between Subject 1 and Subject 2 for the respective events. To reflect dyadic performance rather than individual variability, the values were converted to absolute differences. For both grasp time difference and start time difference, smaller values indicate higher levels of synchrony. A visual inspection of the distributions for these measures revealed the presence of extreme values, likely due to momentary distractions by participants. To address this, we removed outliers using Tukey's interquartile range method (Dhana 2018). The resulting ranges for grasp time difference and start time difference were 33–266.6 ms and 31.3–557.1 ms, respectively. Finally, values were averaged to produce one data point per block, resulting in a total of 16 data points per dyad. Descriptive statistics for behavioral variables are presented in Table 2.

**TABLE 2:** Continuous predictors—means and standard deviations of the continuous predictors included in the models.

	Mean (SD)
Grasp time difference	112.4 (35.9)
Start time difference	165.3 (78.6)
Perspective taking (individual)	27.8 (3.8)
Social anxiety (individual)	38.2 (20.7)
Perspective taking (sum)	55.7 (4.9)
Social anxiety (sum)	76.4 (25.3)
HRV change (sum)	−0.24 (6.5)

### 2.12 Analytical Approach

RQ1 was investigated at the dyad level using mixed‐effects linear models that included both between‐dyad and within‐dyad predictors. Between‐dyad variables, such as block type, block novelty, grasp time difference, and start time difference, are those where both members of the dyad have identical scores. In contrast, within‐dyad variables, such as social anxiety, perspective taking, and HRV change, vary between the two dyad members. Because synchrony is a between‐dyad outcome, a specialized approach (Kenny 2023) was used to estimate the effects of within‐dyad variables on between‐dyad outcomes. This approach involves several steps. First, a full or “saturated” model is computed, including both the sum and the absolute difference of the two members' scores for each within‐dyad predictor. The absolute difference scores are included to account for the possibility that the two scores influence the dependent variable in a nonlinear manner, where the higher score might have a stronger effect than the lower score. If none of the absolute difference predictors significantly affect the outcome, they are removed, and a simplified or “trimmed” model is computed, retaining only the sum scores to estimate the effects. The models included a by‐dyad random intercept and as many random slopes as possible without causing convergence issues. Fixed‐effect estimates, confidence intervals (CIs), and *p*‐values were calculated using parametric bootstrapping with 9999 repetitions, and the *p*‐values were further adjusted using the false discovery rate (FDR) method to control for multiple comparisons. Bootstrapping was conducted using the *boot_summary* function in *R*. Mixed models *R*‐squared (conditional and marginal) was computed with the r2_nakagawa function in *R*. Moreover, each model was compared to a null model that included only the dependent variable and a by‐dyad random intercept using the *anova* function in *R*. This comparison was performed to assess whether the selected predictors collectively explained a significant portion of the variation in the dependent variable.

RQ2 and RQ3 were investigated at the subject level using the method suggested by West (2013) and Kenny, Kashy, and Cook (2020). Specifically, for each dependent variable (HRV change, Performance goodness, Easiness, Pleasantness, Same wavelength, and IOS), we built a two‐level crossed model, with participants nested within dyads and repeated measures crossed with participants. We also included the maximal number of random slopes permitted by the models and estimated CIs and *p*‐values with bootstrapping, as we did for the between‐dyad models. Means and standard deviations of the continuous predictors included in the models are presented in Table 2.

## 3 Results

### 3.1 Control Analysis: Interpersonal Physiological Synchrony in Baseline Versus Task and Real Versus Pseudo Dyads

We compared the distribution of physiological synchrony values obtained during the joint action task with those obtained during the baseline, where no interpersonal synchronization should be observed, given that the dyad members were not interacting. Indeed, a Welch's t‐test for unequal samples (*t* = −4.143, df = 46.17, *p* < 0.001, 95% CIs = [−0.006, −0.0021]) indicates that physiological synchrony values were higher in the joint action task (*M* = 0.0040, SD = 0.007) than in the baseline block (*M* = −0.00007, SD = 0.006), see Figure 2b (right plot). For the baseline condition, the PSEUDO‐REAL comparison was nonsignificant (*t* = −0.158, df = 41.424, *p* = 0.87, 95% CIs = [−0.0021, 0.0017]), while for the joint action one, we found that physiological synchrony values were significantly higher in the REAL (*M* = 0.0001, SD = 0.0071) compared to the PSEUDO (*M* = 0.0040, SD = 0.0062) distribution (*t* = −13.32, df = 689.73, *p* < 0.0001, 95% CIs [−0.0044, −0.0032]), see Figure 2b (right plot). Thus, the physiological synchrony measured from our participants during the joint action task was significantly higher than both the synchrony occurring by chance and the synchrony observed during a noninteractive moment from the same dyads.

### 3.2 Behavioral Synchrony

The analyses examining the impact of contextual and individual aspects on behavioral synchrony (grasp time difference and start time difference) are presented in Supporting Information (see Models S1 and S2 and Figure S6).

### 3.3 RQ1: Which Factors Promote the Emergence of Physiological Synchrony During Joint Action?

We estimated the role of Block Type (free vs. leader–follower), Block Novelty, Grasp time difference, Start time difference, Social Anxiety, Perspective Taking, and HRV change on Physiological Synchrony. To make the model estimates more understandable since physiological synchrony values were very low (min = −0.0189, max = 0.0252, *M* = 0.0040), we multiplied them by 100. As Grasp time difference and Start time difference were significantly correlated (*r* = 0.39, *p* = 0.014), to address potential multicollinearity issues, we investigated the impact of these variables in two separate models. In the “saturated” models, the absolute difference predictors failed to show any significant effect on the outcome (see the “Analytical Approach” section for details) and were discarded. The trimmed model (Model 1) only included the sum values of the within‐dyad predictors.

Model 1

$$\begin{array}{c} \text{Physiological}\;{\text{Synchrony}}_{\mathrm{ij}}=\beta_{0}+\beta_{1}\cdot \text{Block}\;{\text{Type}}_{i}\\ +\beta_{2}\cdot \text{Block}\;{\text{Novelty}}_{i}+\beta_{3}\cdot \text{SUM HRV}\;{\text{change}}_{i}\\ +\beta_{4}\cdot \mathrm{SUM}\;\text{Social}\;{\text{Anxiety}}_{i}+\beta_{5}\cdot \mathrm{SUM}\;\text{Perspective}\;{\text{taking}}_{i}\\ +\beta_{6}\cdot \text{Grasp Time}\;{\text{Difference}}_{i}+b_{0j}+b_{1j}\cdot \text{Block}\;{\text{Type}}_{i}+\epsilon_{\mathrm{ij}} \end{array}$$

**FIGURE 3:**
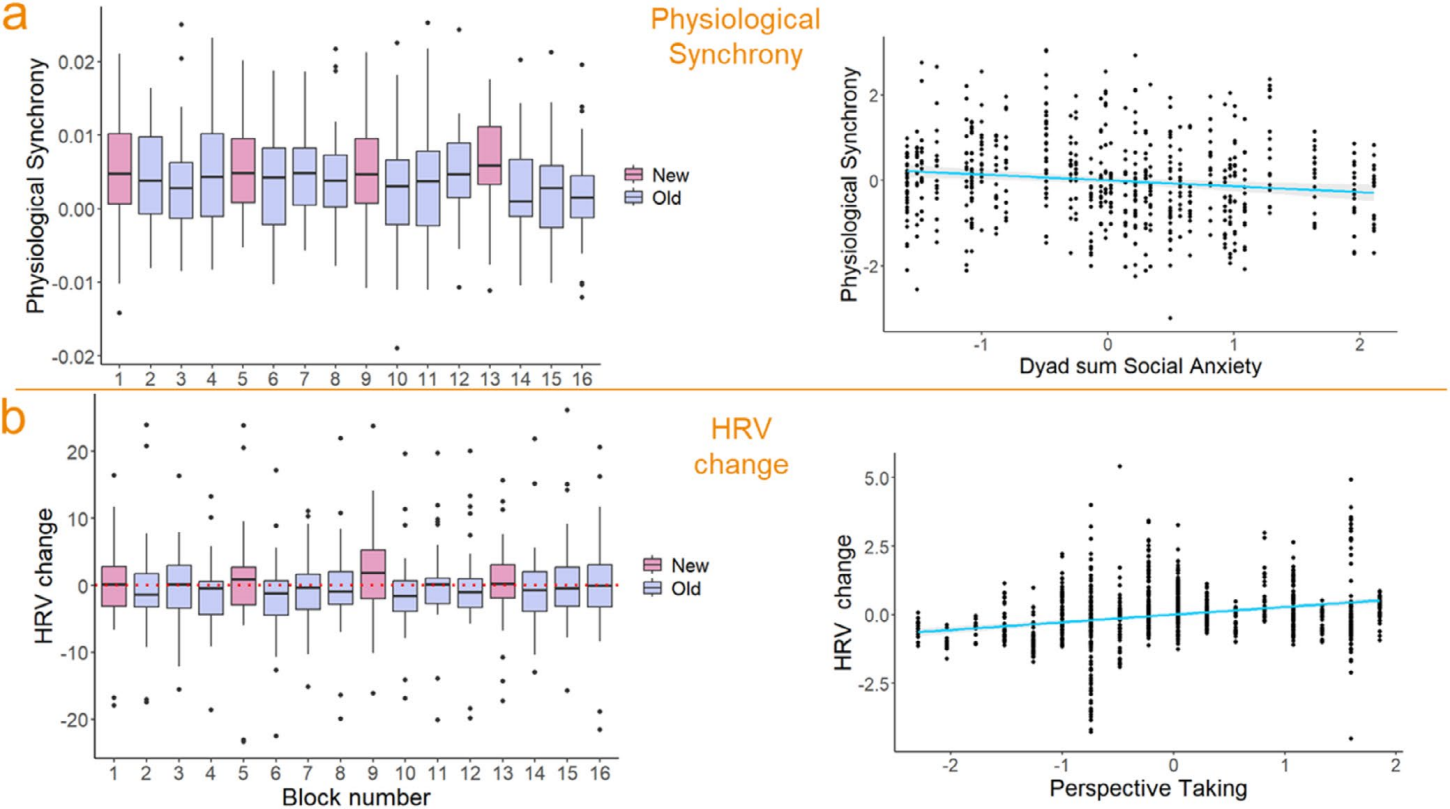
Physiological results. Panel (a) Significant effect of block novelty and dyad‐level social anxiety on physiological synchrony. Panel (b) Significant effects of block novelty and perspective taking on baseline‐corrected HRV individual values. Continuous variables are mean centered to facilitate visualization.

Model 1 (*R* ^2^_conditional_ = 0.210, *R* ^2^_marginal_ = 0.062, AIC = 1216.0) significantly outperformed the null model (*R* ^2^_conditional_ = 0.126, *R* ^2^_marginal_ = 0.00, AIC = 1233.9, *X* ^2^ = 33.65, df = 8, *p* < 0.0001). There was a significant effect of Block Novelty (*b* = −0.187, CI [−0.306, −0.069], *p* =. 005), indicating that physiological synchrony values were higher in new (*M* = 0.55, SD = 0.71) compared to old (*M* = 0.35, SD = 0.71) blocks (see Figure 3a, left plot). In addition, there was a significant effect of Social Anxiety (*β* = −0.20, CI [−0.225, −0.053], *p* = 0.005), indicating a negative relationship between social anxiety and physiological synchrony (see Figure 3a right plot). None of the other predictors were significant (see Supporting Information for full model details). No significant relationship was observed between Grasp time difference and Physiological Synchrony (*b* = 0.042, CI [−0.017, 0.102], *p* = 0.23). When replacing grasp time difference with start time difference, we again observed significant effects of block novelty and social anxiety (see Supporting Information for full model results) and no significant effect of start time difference (*β* = 0.009, CI [−0.06, 0.078], *p* = 0.80). This analysis identified block novelty and social anxiety as significant predictors of physiological synchrony. Specifically, while more socially anxious dyads were less able to synchronize at the physiological level, synchrony increased when participants switched to a new version of the task.

However, since “new” blocks were novel for both participants, we wanted to rule out the possibility that the observed effect was due to the shared context. To this end, we ran a control analysis using the Physiological Synchrony values calculated from pseudo dyads. The model included the fixed effects of Block Type, Block Novelty, and a by‐pseudo dyad random intercept. The effect of Block Novelty was nonsignificant (*b* = 0.000071, *p* = 0.591), see Figure S5, indicating that the observed effect of Block Novelty on Physiological Synchrony was a truly social phenomenon. None of the other predictors were significant.

### 3.4 RQ2: Are Task‐Related Changes in HRV Modulated by Contextual and Individual Factors?

This analysis evaluates the impact of Block Type, Block Novelty, Social Anxiety, and Perspective Taking on baseline‐corrected RMSSD values (HRV change).

Model 2

$$\begin{array}{c} \mathrm{HRV}\;{\text{change}}_{\mathrm{ij}}=\beta_{0}+\beta_{1}\cdot \text{Block}\;{\text{Type}}_{i}\\ +\beta_{2}\cdot \text{Block}\;{\text{Novelty}}_{i}+\beta_{3}\cdot \text{Social}\;{\text{Anxiety}}_{i}\\ +\beta_{4}\cdot \text{Perspective}\;{\text{taking}}_{i}+b_{0j}\\ +b_{1j}\cdot \text{Block}\;{\text{Novelty}}_{i}+b_{2k}+\epsilon_{\mathrm{ijk}} \end{array}$$

Model 2 (*R* ^2^_conditional_ = 0.718, *R* ^2^_marginal_ = 0.080, AIC = 1216.0) significantly outperformed the null model (*R* ^2^_conditional_ = 0.126, *R* ^2^_marginal_ = 0.00, AIC = 1233.9, *X* ^2^ = 33.65, df = 8, *p* < 0.0001). The model revealed a significant effect of Block Novelty (*b* = −0.58, CI [−0.998, −0.167], *p* = 0.013), with higher HRV change values in new compared to old blocks, see Figure 3b, left plot, and Table 1. We also found a significant (inverse) effect of Perspective Taking on HRV change (*β* = 1.23, CI [0.682, 1.789], *p* < 0.0001), see Figure 3b, right plot. All other predictors were nonsignificant (see Supporting Information for full model results and Table 1 for descriptive statistics). Overall, this analysis indicates that baseline‐corrected participants' RMSSD increased in “new” task blocks compared to repetition, and that RMSSD values increased with participants' perspective taking abilities.

### 3.5 RQ3: Which Factors Influence the Subjective Experience of the Interaction?

**FIGURE 4:**
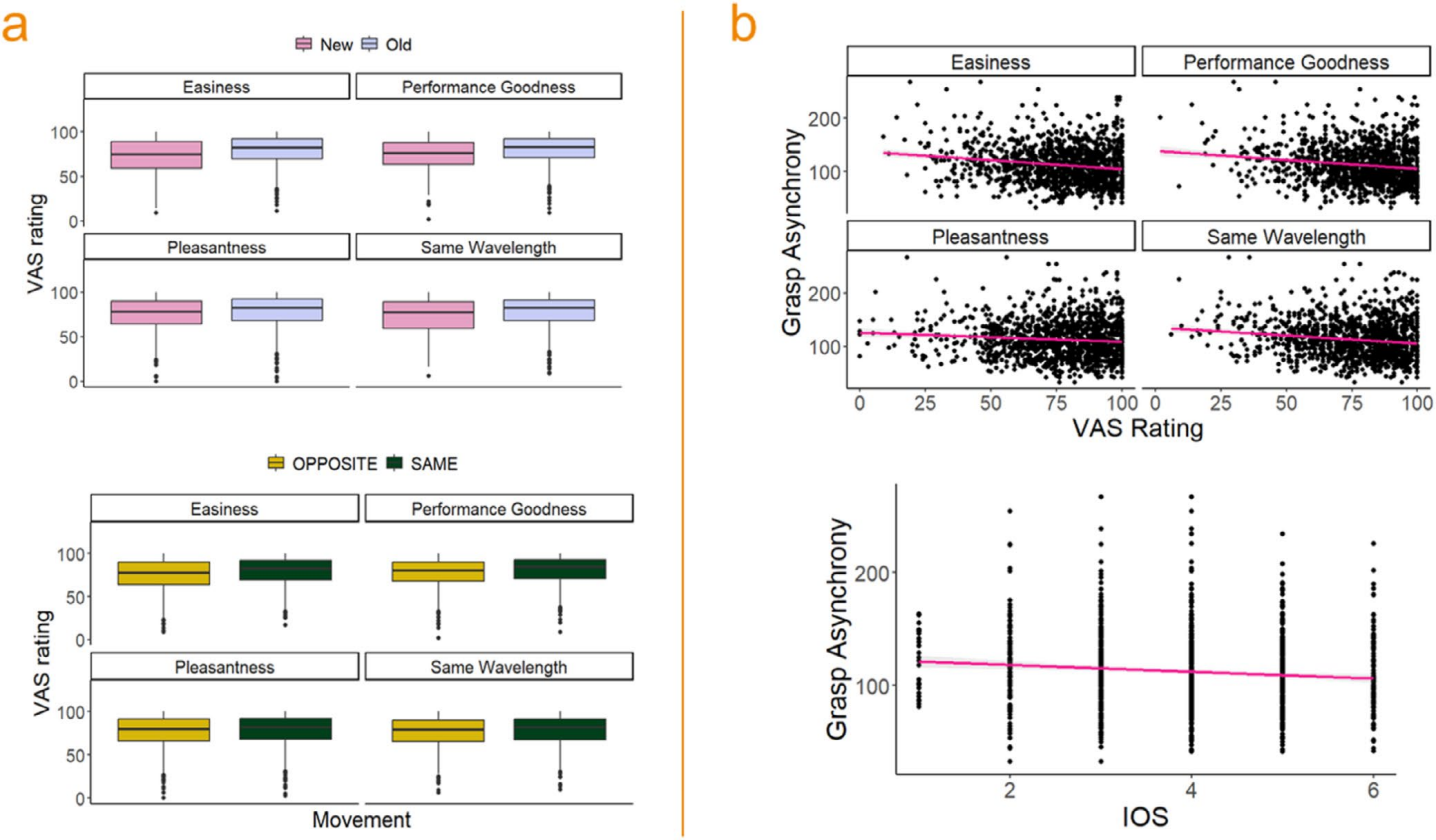
Subjective reports results—Panel (a) Significant effects of block novelty (upper plot) and movement (lower plot) on subjective feelings collected at the end of each block. Panel (b) Significant effects of grasping asynchrony on subjective feelings (upper plot) and IOS scores (lower plot).

We estimated the role of Block Type, Movement (same vs. opposite), Block Novelty, Social Anxiety, Perspective Taking, Grasp time difference, and Physiological Synchrony on each item of the Subjective Experience questionnaire items; see Figure 4. Each item was analyzed individually using mixed‐effects models with the same model structure (see Model 3).

Model 3

$$\begin{array}{c} \text{Subjective}\;{\text{experience}}_{\mathrm{ij}}=\beta_{0}+\beta_{1}\cdot {\text{Movement}}_{i}\\ +\beta_{2}\cdot \text{Block}\;{\text{Type}}_{i}+\beta_{3}\cdot \text{Block}\;{\text{Novelty}}_{i}\\ +\beta_{4}\cdot \text{Physiological}\;{\text{Synchrony}}_{i}+\beta_{5}\cdot \text{Social}\;{\text{Anxiety}}_{i}\\ +\beta_{6}\cdot \text{Perspective}\_{\text{taking}}_{i}+\beta_{7}\cdot \text{Grasp Time}\;{\text{Difference}}_{i}\\ +\beta_{8}\cdot \mathrm{HRV}\;{\text{change}}_{i}\;b_{0j}+b_{1j}\cdot \text{Block}\;{\text{Novelty}}_{i}+b_{2k}+\epsilon_{\mathrm{ijk}} \end{array}$$

The model for Performance goodness (*R* ^2^_conditional_ = 0.61, *R* ^2^_marginal_ = 0.07, AIC = 9139.3) significantly outperformed the null model (*R* ^2^_conditional_ = 0.55, *R* ^2^_marginal_ = 0.00, AIC = 9250.7, *X* ^2^ = 129.44, df = 9, *p* < 0.0001). Participants perceived their dyadic performance as better in same compared to opposite blocks (*b* = 2.39, CI [1.17, 3.60], *p* < 0.001) and in old compared to new blocks (*b* = 5.32, CI [3.79, 6.82], *p* < 0.001). Performance goodness was negatively associated with grasp time difference (*β* = −2.45, CI [−3.2, −1.69], *p* < 0.001). Thus, perceived performance increased as actual interpersonal performance improved. Concerning Easiness, the model (*R* ^2^_conditional_ = 0.59, *R* ^2^_marginal_ = 0.09, AIC = 9309.3) showed a better fit compared to the null (*R* ^2^_conditional_ = 0.51, *R* ^2^_marginal_ = 0.00, AIC = 9449.1, *X* ^2^ = 78.69, df = 9, *p* < 0.0001). Participants rated the task as easier in same compared to opposite (*b* = 2.792, CI [1.447, 4.126], *p* < 0.001), in old versus new blocks (*b* = 6.071, CI [4.141, 7.955], *p* < 0.001), and in leader–follower compared to free blocks (*b* = 3.953, CI [0.875, 7.092], *p* = 0.018), see Figure 4 and Table 1. Easiness was negatively associated with grasp time difference (*β* = −3.121, CI [−3.967, −2.289], *p* < 0.001). All the other predictors in both models were nonsignificant (see Supporting Information for the full model results).

The Pleasantness model (*R* ^2^_conditional_ = 0.76, *R* ^2^_marginal_ = 0.058, AIC = 9057.0) showed a better fit compared to the null (*R* ^2^_conditional_ = 0.732, *R* ^2^_marginal_ = 0.00, AIC = 9117.7, *X* ^2^ = 78.69, df = 9, *p* < 0.0001). Participants rated the task as more pleasant in same compared to opposite blocks (*b* = 1.44, CI [0.307, 2.572], *p* = 0.021) and in old compared to new blocks (*b* = 2.701, CI [1.112, 4.274], *p* = 0.001). Perceived pleasantness of the interaction was negatively related to social anxiety (*β* = −3.80, CI [−6.153, −1.376], *p* = 0.003), indicating that more socially anxious participants enjoyed the interaction less. Perceived pleasantness was also negatively related to Grasp Time Difference (*β* = −2.085, CI [−2.813, −1.364], *p* < 0.001). Similarly, for Same wavelength, the model (*R* ^2^_conditional_ = 0.64, *R* ^2^_marginal_ = 0.06, AIC = 9224.9) outperformed the null (*R* ^2^_conditional_ = 0.57, *R* ^2^_marginal_ = 0.00, AIC = 9332.9, *X* ^2^ = 129.44, df = 9, *p* < 0.0001). Participants felt more on the same wavelength with the partner in same compared to opposite blocks (*b* = 2.02, CI [0.741, 3.289], *p* = 0.003) and in old compared to new blocks (*b* = 4.280, CI [2.269, 6.236], *p* < 0.001). Ratings of being on the same wavelength were negatively related to grasp time difference (*β* = −3.10, CI [−3.91, −2.31], *p* < 0.001), see Figure 4. All the other predictors in both models were nonsignificant.

#### 3.5.1 Inclusion of the Other in the Self (IOS)

Responses to the IOS questionnaire were given on a 6‐point Likert scale; thus, we analyzed them using cumulative link mixed models fitted with the *clmm* function in the R package “ordinal” (Christensen 2022). Our model evaluated the impact of Block Type, Movement, Physiological Synchrony, Grasp time difference, Social Anxiety, and Perspective Taking on IOS responses. The model (*R* ^2^_conditional_ = 0.86, *R* ^2^_marginal_ = 0.016, AIC = 2341.9) revealed a significant effect of Movement (*b* = 0.345, std. error = 0.12, *z* value = 2.67, *p* < 0.001), indicating that participants felt closer to their partner in same (*M* = 4.05, SD = 1.22) than in opposite (*M* = 3.91, SD = 1.25) blocks. Moreover, a significant effect of Grasp time difference (*β* = −0.341, std. error = 0.08, *z* value = −4.07, *p* < 0.0001) revealed that, as performance improved (smaller Grasp Time Difference values), participants felt more close to their partner; see Figure 4b. All other predictors were nonsignificant (see Supporting Information for full model results).

## 4 Discussion

**FIGURE 5:**
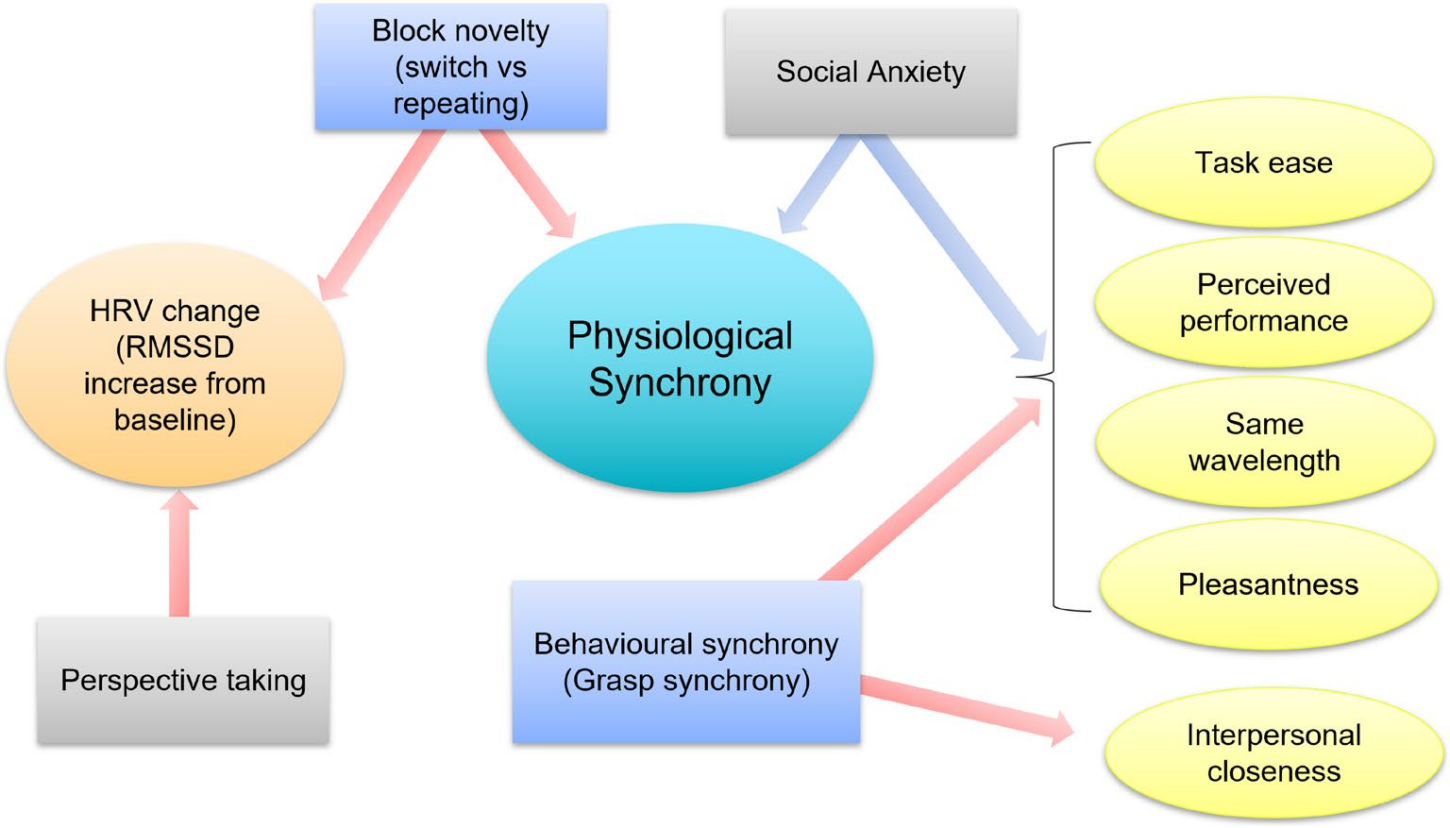
The effects of contextual (blue squares) and individual factors (gray squares) on physiological and subjective dependent variables (ovals). Arrows represent the direction of influence in the regressions, moving from the independent variables to the dependent variables. Red arrows indicate a significant positive effect, while blue arrows indicate a significant negative effect. It is important to note that the figure does not represent a single integrated model, such as in structural equation modeling. Instead, it serves as a visual summary of the main findings, with most relationships analyzed independently.

This study explored the emergence of physiological synchrony during joint action. We observed that physiological synchrony was significantly greater during the joint grasping task compared to a noninteractive baseline task and synchrony occurring by chance. We then examined the contextual and individual factors that predicted physiological synchrony, as well as task‐induced changes in HRV and subjective experiences. A graphical summary of these findings is presented in Figure 5.

### 4.1 Switching to a New Task Increases Physiological Synchrony and HRV

Our main finding was that physiological synchrony increased whenever the dyad switched to a slightly different version of the joint grasping task, suggesting that the need to learn something new together with another person may enhance the interpersonal synchronization of physiological states. One possible explanation for this result is that new blocks, being more challenging and requiring close monitoring of each other's actions (Vesper et al. 2010), drove participants into a state of reciprocal attention that facilitated physiological synchrony. The link between task difficulty and physiological synchrony is in line with Malmberg and colleagues' study (Malmberg et al. 2019), where, in groups of four participants engaged in a collaborative exam, synchrony in EDA was observed only in teams that experienced difficulties during the task. Similarly, physiological synchrony was found to be related to group levels of collective mental effort (Dindar et al. 2020), while a positive relationship was found between physiological synchrony in presenters and audience members during a conference and dyad‐level self‐reported engagement (Gashi et al. 2019). Other similar results were observed when measuring physiological synchrony in friends (Slovak et al. 2014) and child–parent (Hernandez et al. 2014) dyads. Additional evidence comes from a seminal study measuring physiological synchrony between firewalkers and the audience during a live performance (Konvalinka et al. 2014). The authors observed that interpersonal cardiac synchrony occurred between performer–spectator dyads that were already related (i.e., relatives or spouses) but not in nonrelated dyads, suggesting that the degree of involvement experienced by the spectators determined the occurrence of physiological synchrony. In addition, inter‐subject correlation in the cardiac activity of individuals attending to the same narrative stimuli was found to reflect attentional engagement (Pérez et al. 2021; Stuldreher et al. 2020). Furthermore, it should be noted that – in our study – “new” blocks contained more information to be processed by the participants. Novel stimuli often necessitate enhanced attentional allocation because they require greater cognitive and sensory processing to extract relevant cues and reduce uncertainty. Overall, these results indicate that physiological synchrony might be a marker of reciprocal attention and engagement.

Another partially related mechanism at play may be that of interpersonal performance monitoring. The joint grasping task requires participants to continuously monitor each other's actions (see Boukarras et al. 2022; Moreau et al. 2020) to predict and understand the partner's goal and synchronize the grasping time. Although we did not explicitly test this hypothesis, it is possible that, after many repetitions of the same task, predicting the actions of the partner become easier and reciprocal action monitoring become less crucial for task execution, thus reducing physiological synchrony. Conversely, changing the task's rules may have forced participants to reactivate the (social) performance monitoring system (Moreau et al. 2023) to face the challenge of the new task demands. This, in turn, may boost the synchronization of cardiac activity, possibly by driving dyad members into a similar state (i.e., a “social monitoring” state). Incidentally, it should be noted that although participants reported the impression that their performance decreased during “new” blocks (see Supporting Information), their grasping asynchrony was comparable to “old” blocks, hinting at a functional role of physiological synchrony in maintaining optimal levels of dyadic performance in a challenging environment.

New blocks were also characterized by a slight increase in baseline‐corrected RMSSD values at the subject level compared to repetitions, although the observed effect was small. Previous studies have reported that increases in HRV during social interactions are positively associated with engagement and attentiveness (Williams et al. 2009; Cribbet et al. 2011), while HRV decreases during rumination (Era et al. 2021). From this perspective, the observed increase in HRV in new blocks may represent, along with the increase in physiological synchrony, another independent marker of social engagement. Another intriguing possibility, although highly speculative, is that the observed increase in HRV in the “new” blocks of our task may be linked to the activation of the performance monitoring system, elicited by the need to deal with the new task demands. Indeed, previous studies found a direct relationship between midfrontal theta oscillations (a neural index of performance monitoring, see Boukarras et al. 2022; Cavanagh and Frank 2014; Moreau et al. 2020) and phasic increases in HRV during meditation tasks (Kubota et al. 2001; Tang et al. 2009). However, further studies combining neural and autonomic data are needed to clarify this aspect.

### 4.2 Physiological Synchrony Is Negatively Associated With Social Anxiety

Concerning the role of individual differences, we found a negative relationship between physiological synchrony and dyad‐level social anxiety, indicating that more socially anxious dyads showed lower levels of physiological synchrony, which is in line with previous studies using conversations (Asher et al. 2021) and group drumming (Gordon et al. 2023). Using a dedicated approach for dyadic data (Kenny 2023), we established that the effect was driven by both dyad members' social anxiety levels, similar to what Gordon and colleagues observed in triads (Gordon et al. 2023). In their work, Gordon et al. (2023) postulate three possible mechanisms for the observed reduction of physiological synchrony in social anxiety: internally focused attention, the use of concealing strategies (e.g., avoiding eye contact), and a misattunement toward affiliative signals. While we agree with the authors that all three putative mechanisms are equally valid and not mutually exclusive, in light of our results, we find the attentional hypothesis particularly intriguing. Studies indicate that socially anxious individuals pay more attention to their internal bodily sensations, resulting in enhanced interoceptive sensibility (Terasawa et al. 2013) and accuracy (Stevens et al. 2011; see Garfinkel et al. 2015, for the distinction between facets of interoception). It is conceivable that this inward focus of attention deprives individuals of the resources needed to process social stimuli and monitor the actions of others. In turn, because physiological synchrony seems to emerge from social attention and engagement, aberrant self‐referenced attention may undermine the conditions necessary for physiological synchrony to occur. At the subject level, social anxiety also negatively predicted self‐reported enjoyment of the interaction, indicating that socially anxious participants may have experienced the task as stressful. Overall, our results suggest that people who tend to avoid and fear social interaction might be less able (or willing) to attune to each other, which may manifest in lower levels of autonomic synchrony.

### 4.3 No Relation Between Behavioral and Physiological Synchrony

We hypothesized a positive relationship between physiological synchrony and task performance, although we did not have a clear expectation regarding which one of the two would influence the other. Specifically, we hypothesized that, either through cardiac synchrony, participants would align their sensorimotor states, thus facilitating behavioral synchrony, or the sensorimotor alignment would strengthen physiological synchrony. Contrary to our expectations, we found no evidence that physiological synchrony was associated with performance, neither for “instructed” (i.e., grasp time difference) nor for “spontaneous” (i.e., start time difference) synchrony indices. It is possible that our sample size did not provide enough power to detect very small effects; thus, our negative findings should not be taken as evidence for the absence of an effect. In particular, because the relationship between physiological and behavioral synchrony is expected to produce very small effect sizes, larger samples could be required to observe a meaningful effect. Furthermore, a recent meta‐analysis reported strong heterogeneity in the effect sizes of the relationship between physiological synchrony and performance in dyadic/group tasks (Mayo et al. 2021). While some studies found a positive relationship, others reported no relation between the two, or even a detrimental effect of physiological synchrony on task performance (see also Algumaei et al. 2023). This heterogeneity may be partly due to intrinsic differences in the types of tasks used and how performance is operationally defined. In our study, the task goal was to synchronize the grasping timing with the partner. Previous studies reported a positive relationship between physiological synchrony and behavioral synchrony during group drumming (Gordon et al. 2020) and the Mirror Game (Noy et al. 2015). However, unlike paradigms where participants perform continuous joint actions, as in ensemble music production or the Mirror Game, the joint grasping task encompasses brief trials (start‐move‐grasp‐return) spaced by brief intertrial intervals. Thus, the actual length of the joint movements might be too short to be influenced by (or to influence) physiological synchrony. Despite this limitation, we still observed above‐chance levels of physiological synchrony during the task, suggesting that behavioral alignment is unlikely to be the only process driving the emergence of autonomic synchrony.

### 4.4 Positive Feelings Associated With the Interaction Are Reduced by Task Novelty and Increase With Behavioral Synchrony

Through subjective experience sampling, we aimed to investigate the phenomenological correlates of physiological synchrony. Subjective reports collected at the end of each block indicated that participants experienced the “new” blocks as less easy, less pleasant, and characterized by reduced dyadic performance and a decreased feeling of “being on the same wavelength.” The finding is apparently at odds with the results reported by Ravreby and colleagues (2022), where they observed that participants' enjoyment of Mirror Game blocks was better explained by a combination of synchrony and novelty than by synchrony alone. This discrepancy possibly highlights an important difference between highly structured, rule‐based tasks and more creative types of joint action in which participants are free to move as they want. In rule‐based tasks such as our joint grasping, the subjective enjoyment of the interaction might arise from the perception of having achieved a good dyadic performance, while in the Mirror Game the pleasure derived from creatively inventing new movements might overshadow the pleasure for synchronous actions. A questionnaire measuring perceived closeness and overlap with the other (IOS, Aron et al. 1992) revealed that participants felt closer to each other in the peer‐to‐peer (free) interaction compared to the leader–follower one. Moreover, IOS values were negatively related to grasp time difference, corroborating the notion that behavioral synchrony increases self‐other overlap and promotes affiliation (Hove and Risen 2009). No relationship was found between perceived performance, task ease, pleasantness or the feeling of “being in the same wavelength,” and physiological synchrony. This indicates that, in our setting, physiological synchrony did not translate at the subjective level neither into feelings of alignment nor into a pleasant experience. The fact that participants were not aware of being synchronized in their cardiac activities (or, at least, they would not express such feeling as “being in the same wavelength”) fits the prediction formulated by Gallotti et al. (2017) regarding joint actions that “[…] what matters for the relevant alignment of minds and bodies to occur is the reciprocal exchange of information, not awareness of the reciprocal exchange of information.” Thus, physiological alignment may emerge from reciprocal action monitoring and prediction without necessarily giving rise to the subjective awareness of “being in sync.”

### 4.5 Limitations and Future Perspectives

This study presents some limitations. First, our conclusions on the role of attention and performance monitoring in physiological synchrony are based on the observation that physiological synchrony increased in blocks requiring increased attention, but we did not collect any measure tackling attentional functions. Future studies may provide additional evidence by including behavioral and/or neural measures of attention and monitoring in studies measuring physiological synchrony during social interactions. Second, while we chose to quantify physiological synchrony using cross‐correlation analysis, multiple statistical methods can be and have been used in the literature. However, an agreement on which methods are more appropriate, in which circumstances they should be used, and what conclusions can be drawn from them is lacking. Future research should shed light on these aspects to provide a clearer and more comprehensive account of physiological synchrony. Third, data collection occurred during the COVID‐19 pandemic—a period when social interactions were often perceived as potentially threatening due to the risk of contagion. Moreover, it is important to highlight that participants wore masks during the task, which obscured facial cues that could play a crucial role in the emergence of interpersonal synchrony. These factors may have influenced our findings, underscoring the importance of replicating this study under nonemergency conditions to better understand the generalizability of our results. Finally, although our observed effect sizes were comparably high, the relatively low sample size remains a limitation and warrants caution when generalizing the results to broader populations.

## 5 Conclusion

Our study revealed the presence of physiological synchrony in a joint action task requiring reciprocal action monitoring and prediction. Physiological synchrony increased during task switching, namely, when action monitoring became more crucial for task completion, suggesting that social performance monitoring and/or reciprocal engagement may underlie the emergence of physiological synchrony. In addition, subclinical social anxiety, which is characterized by an aberrant shift of an individual's attentional focus toward the self, hindered the emergence of physiological synchrony and decreased participants' enjoyment of the interaction. Overall, the results indicate that the alignment of physiological states is not merely a byproduct of behavioral alignment or shared task demands, but rather that it may reflect processes of reciprocal attention and monitoring that are fundamental to joint action. While the set of potential predictors we put to test in our work is far from exhaustive, our study advances the current understanding of physiological synchrony in joint action by highlighting the pivotal influence of contextual factors and the individual contributions of each participant, including their abilities and willingness to engage in interaction.

## Author Contributions

**Sarah Boukarras:** conceptualization, data curation, formal analysis, investigation, methodology, project administration, writing – original draft, writing – review and editing. **Valerio Placidi:** data curation, investigation, project administration, writing – review and editing. **Federico Rossano:** data curation, formal analysis, methodology, writing – review and editing. **Vanessa Era:** conceptualization, project administration, writing – review and editing. **Salvatore Maria Aglioti:** conceptualization, supervision, writing – review and editing. **Matteo Candidi:** conceptualization, funding acquisition, supervision, writing – review and editing.

## Conflicts of Interest

The authors declare no conflicts of interest.

## Supporting information


Data S1.

## Data Availability

The data and codes that support the findings of this study are openly available at the OSF repository, https://osf.io/cbdxf/?view_only=63e86b6cf0c84ad09241d0b9074c472d.
