# Supplementary material for: Interpersonal Physiological Synchrony During Dyadic Joint Action Is Increased by Task Novelty and Reduced by Social Anxiety
Source: Psychophysiology. 2025 Mar 17;62(3):e70031. doi: 10.1111/psyp.70031 (PMC11913774; doi:10.1111/psyp.70031)
Supplement: Supplementary file 1 — Data S1. [file PSYP-62-e70031-s001.pdf]

Supplementary Materials

Interpersonal physiological synchrony during dyadic joint action is increased by task novelty and reduced by social anxiety

Sarah Boukarras[1,2], Valerio Placidi[1,3,4], Federico Rossano[1,2,4], Vanessa Era[1,2], Salvatore Maria Aglioti[1,2,4] & Matteo Candidi[1,2]

1 Department of Psychology, Sapienza University of Rome, Italy
2 Santa Lucia Foundation (IRCCS), Rome, Italy
3 University of Camerino, Centre for Neuroscience, Camerino, Italy
4 Sapienza University of Rome and CLN2S@Sapienza, Italian Institute of Technology, Rome, Italy

1- Control analysis on CCF distribution across lags

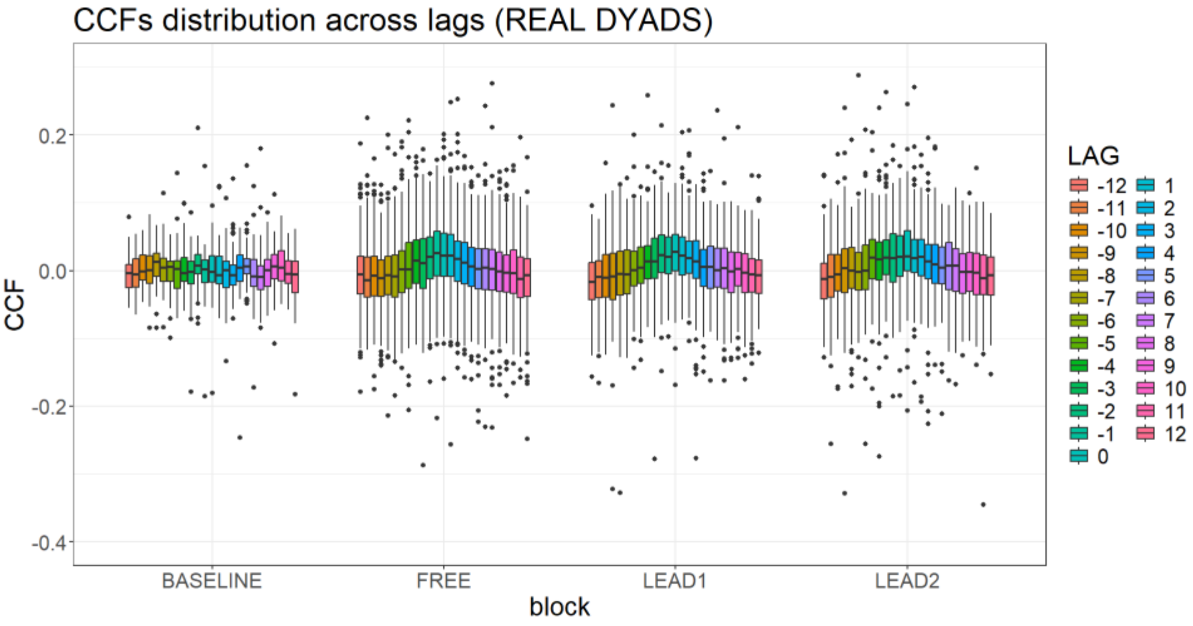

Figure S1 - Distribution of cross-correlation coefficients from real dyads across lags (from -12 to 12). Note that IBI data were sampled at 4 Hz, thus groups of four consecutive lags correspond to 1 second (e.g. from -12 to -9 corresponds to -3 seconds).

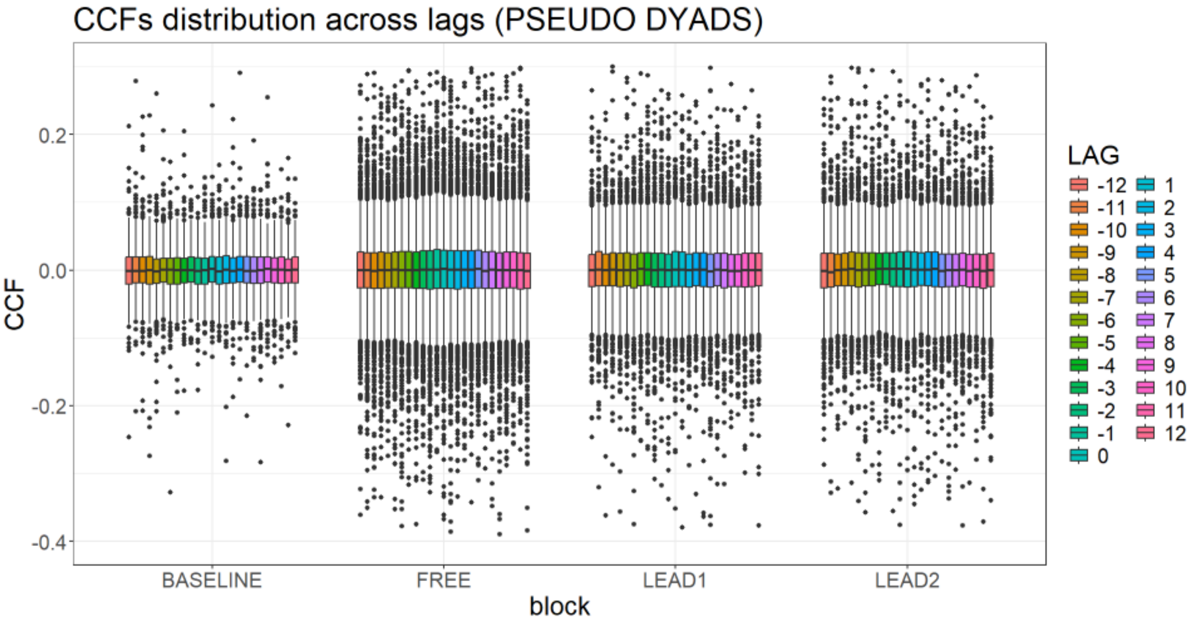

Figure S2 - Distribution of cross-correlation coefficients from spurious (PSEUDO) dyads (N = 13.260) across lags (from -12 to 12). Note that IBI data were sampled at 4 Hz, thus groups of four consecutive lags correspond to 1 second (e.g. from -12 to -9 corresponds to -3 seconds).

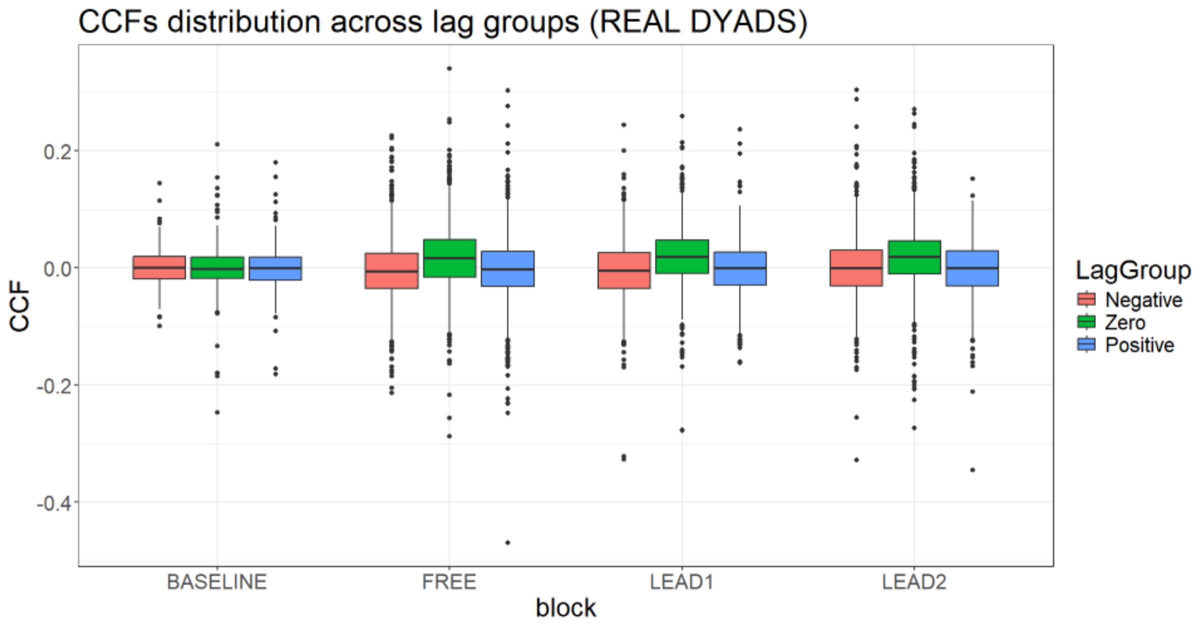

Figure S3 - Distribution of cross-correlation coefficients averaged over lag groups : “Zero” (from lag -4 to lag 4), “Negative” (from lag -12 to lag -3) and “Positive” (from lag 5 to lag 12).

Model on CCF values averaged at Zero lag group

Full model structure:

PhysiologicalSynchrony_ZEROlag ~ block_type+movement+block novelty +
scale(SUM_HRV)+
scale(SUM_Social_anxiety)+
scale(SUM_Perspective_taking)+
scale(Grasp_asy)+
(1|dyad)

Full results table:

group		Estimate	Standard Error	df	statistic	p-value	
		Fixed effects					
	(Intercept)	0.019	0.002	122	7.997	0.0000	***
	block_typeLEADER-FOLLOWER	0.001	0.002	598	0.922	0.3567	
	movementSAME	0.001	0.001	596	0.542	0.5883	
	old_newOld	-0.005	0.002	601	-2.619	0.0090	**
	scale(SUM_HRV)	-0.002	0.001	99	-1.519	0.1320	
	scale(SUM_Social_anxiety)	-0.001	0.002	36	-0.393	0.6967	
	scale(SUM_Perspective_taking)	-0.000	0.002	36	-0.085	0.9328	
	scale(Grasp_asy)	0.001	0.001	630	0.872	0.3833	
		Random effects					
dyad	sd__(Intercept)	0.010					
Residual	sd__Observation	0.019					

Signif. codes: 0 <= '***' < 0.001 < '**' < 0.01 < '*' < 0.05

square root of the estimated residual variance: 0.0

data's log-likelihood under the model: 1,557.0

Akaike Information Criterion: -3,094.0

Bayesian Information Criterion: -3,049.3

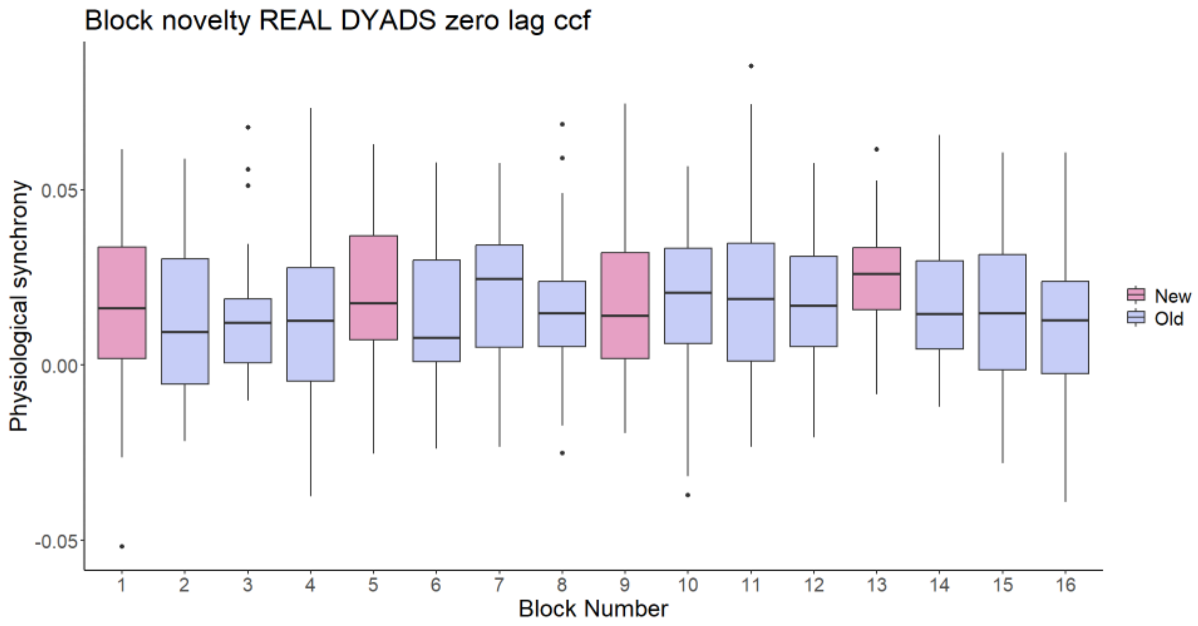

Figure S4 - CCF values averaged over the Zero lag group across blocks.

2- Control analysis on block novelty effect for pseudo dyads

Full model structure:

PhysiologicalSynchrony_PSEUDO ~ block+movement+block novelty + (1|PSdyad)

Full results table:

group		Estimate	Standard Error	df	statistic	p-value
		Fixed effects				
	(Intercept)	0.000	0.000	6,017	0.172	0.8636
	blockLEAD1	0.000	0.000	410	0.389	0.6972
	blockLEAD2	0.000	0.000	411	0.680	0.4969
	movementSAME	0.000	0.000	12,318	1.387	0.1656
	old_newOld	0.000	0.000	3,874	0.537	0.5913
		Random effects				
PSdyad	sd__(Intercept)	0.000				
Residual	sd__Observation	0.006				

Signif. codes: 0 <= '***' < 0.001 < '**' < 0.01 < '*' < 0.05

square root of the estimated residual variance: 0.0

data's log-likelihood under the model: 45,604.0

Akaike Information Criterion: -91,194.0

Bayesian Information Criterion: -91,141.9

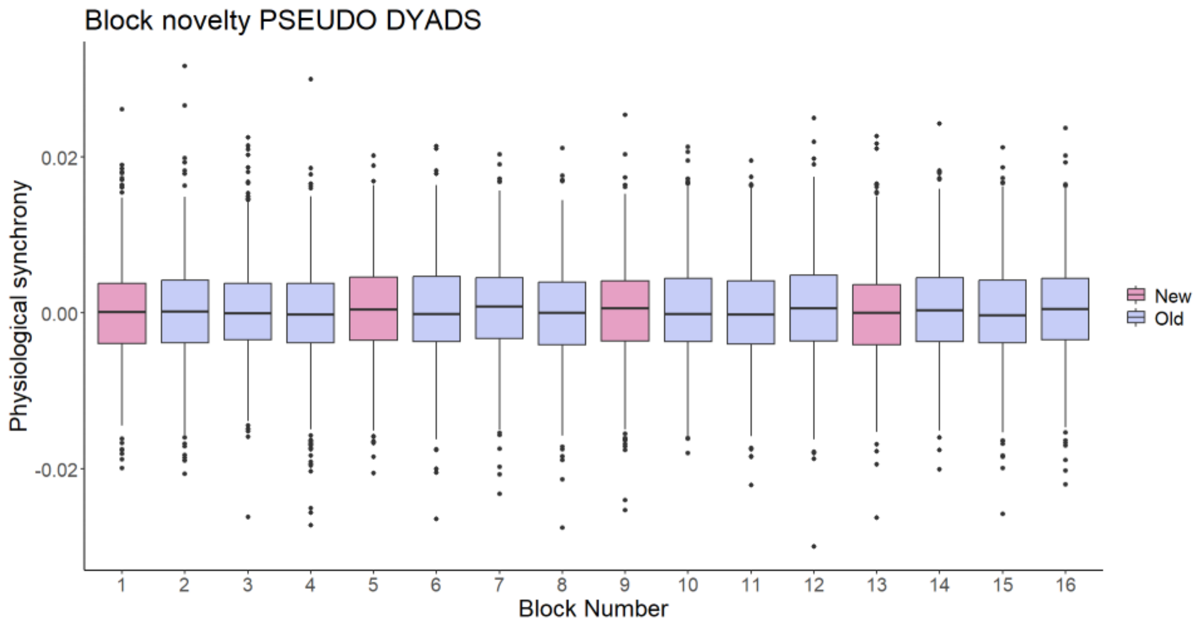

Figure S5 - Average CCF values for pseudo dyads across blocks.

3 - Control analysis checking the interaction between Block Type and Social Anxiety on Physiological Synchrony

group		Estimate	Standard Error	df	statistic	p-value	
	Fixed effects						
	(Intercept)	0.455	0.065	141	6.994	0.0000	***
	Block_noveltyOld	-0.190	0.061	522	-3.117	0.0019	**
	block_typeLEADER-FOLLOWER	0.127	0.072	47	1.775	0.0824	.
	scale(SUM_HRV_change)	-0.019	0.037	139	-0.505	0.6146	
	scale(SUM_Social_anxiety)	-0.166	0.063	154	-2.627	0.0095	**
	scale(SUM_Perspective_taking)	0.017	0.044	40	0.380	0.7056	
	scale(Start_Time_Difference)	0.009	0.035	396	0.249	0.8037	
	Block_noveltyOld:scale(SUM_Social_anxiety)	0.041	0.061	515	0.666	0.5057	
	Random effects						
dyad	sd__(Intercept)	0.157					
dyad	cor__(Intercept).block_typeLEADER-FOLLOWER	0.367					
dyad	sd__block_typeLEADER-FOLLOWER	0.255					
Residual	sd__Observation	0.636					

Signif. codes: 0 <= '***' < 0.001 < '**' < 0.01 < '*' < 0.05

4 - Model tables (following bootstrapping)

Model 1 - DV: Physiological Synchrony

	Estimate	95 % CI	p-value	Adjusted p-value
(Intercept)	0.455	(0.328, 0.578)	0.0000	0.0000
Block_noveltyOld	-0.187	(-0.306, -0.069)	0.0019	0.0051
block_typeLEADER-FOLLOWER	0.126	(-0.006, 0.258)	0.0608	0.1064
scale(SUM_HRV_change)	-0.023	(-0.095, 0.049)	0.5312	0.6191
scale(SUM_Social_anxiety)	-0.139	(-0.225, -0.053)	0.0022	0.0051
scale(SUM_Perspective_taking)	0.022	(-0.064, 0.111)	0.6191	0.6191
scale(Grasp_Time_Difference)	0.042	(-0.017, 0.102)	0.1651	0.2312

Model 1a - DV: Physiological Synchrony, Start Time Difference as a predictor (replacing Grasp Time Difference)

	Estimate	95 % CI	p-value	Adjusted p-value
(Intercept)	0.455	(0.329, 0.581)	0.0000	0.0000
Block_noveltyOld	-0.190	(-0.309, -0.07)	0.0016	0.0037
block_typeLEADER-FOLLOWER	0.127	(-0.015, 0.269)	0.0760	0.1330
scale(SUM_HRV_change)	-0.018	(-0.091, 0.056)	0.6354	0.8047
scale(SUM_Social_anxiety)	-0.136	(-0.223, -0.049)	0.0011	0.0037
scale(SUM_Perspective_taking)	0.017	(-0.073, 0.105)	0.7219	0.8047
scale(Start_Time_Difference)	0.009	(-0.06, 0.078)	0.8047	0.8047

Model 2 DV: Baseline-corrected RMSSD

	Estimate	95 % CI	p-value	Adjusted p-value
(Intercept)	0.249	(-0.788, 1.259)	0.6590	0.7065
block_typeLEADER-FOLLOWER	0.165	(-0.75, 1.096)	0.7065	0.7065
Block_noveltyOld	-0.586	(-0.998, -0.167)	0.0055	0.0138
scale(Social_anxiety)	-0.284	(-0.825, 0.263)	0.3124	0.5207
scale(Perspective_taking)	1.234	(0.682, 1.789)	0.0000	0.0000

Model 3a DV: Performance goodness

	Estimate	95 % CI	p-value	Adjusted p-value
(Intercept)	71.541	(67.749, 75.232)	0.0000	0.0000
movementSAME	2.415	(1.179, 3.654)	0.0000	0.0000
Block_noveltyOld	5.327	(3.791, 6.807)	0.0000	0.0000
block_typeLEADER-FOLLOWER	3.063	(-0.206, 6.359)	0.0695	0.1039
scale(PhysiologicalSynchrony_100)	-0.636	(-1.349, 0.081)	0.0808	0.1039
scale(Social_anxiety)	-1.377	(-3.267, 0.541)	0.1599	0.1799
scale(Perspective_taking)	-1.911	(-3.873, 0.1)	0.0623	0.1039
scale(Grasp_Time_Difference)	-2.453	(-3.249, -1.666)	0.0001	0.0002
scale(HRV_change)	-0.104	(-1.192, 0.984)	0.8443	0.8443

Model 3b DV: Same wavelength

	Estimate	95 % CI	p-value	Adjusted p-value
(Intercept)	71.752	(67.498, 75.875)	0.0000	0.0000
movementSAME	2.042	(0.755, 3.316)	0.0019	0.0043
Block_noveltyOld	4.306	(2.31, 6.245)	0.0000	0.0000
block_typeLEADER-FOLLOWER	1.279	(-2.172, 4.776)	0.4573	0.5145
scale(PhysiologicalSynchrony_100)	-0.392	(-1.129, 0.345)	0.3062	0.3937
scale(Social_anxiety)	-1.981	(-4.001, 0.062)	0.0565	0.1017
scale(Perspective_taking)	-1.532	(-3.643, 0.604)	0.1554	0.2331
scale(Grasp_Time_Difference)	-3.113	(-3.937, -2.298)	0.0001	0.0003
scale(HRV_change)	0.092	(-1.036, 1.219)	0.8734	0.8734

Model 3c DV: Easiness

	Estimate	95 % CI	p-value	Adjusted p-value
(Intercept)	68.852	(64.746, 72.857)	0.0000	0.0000
movementSAME	2.804	(1.444, 4.159)	0.0000	0.0000
Block_noveltyOld	6.010	(4.082, 7.895)	0.0000	0.0000
block_typeLEADER-FOLLOWER	3.953	(0.836, 7.073)	0.0125	0.0225
scale(PhysiologicalSynchrony_100)	-0.662	(-1.433, 0.115)	0.0948	0.1219
scale(Social_anxiety)	-1.912	(-3.732, -0.062)	0.0409	0.0614
scale(Perspective_taking)	-1.452	(-3.355, 0.488)	0.1388	0.1562
scale(Grasp_Time_Difference)	-3.109	(-3.971, -2.253)	0.0001	0.0002
scale(HRV_change)	-0.551	(-1.706, 0.602)	0.3519	0.3519

Model 3d DV: Same wavelength

	Estimate	95 % CI	p-value	Adjusted p-value
(Intercept)	71.752	(67.498, 75.875)	0.0000	0.0000
movementSAME	2.042	(0.755, 3.316)	0.0019	0.0043
Block_noveltyOld	4.306	(2.31, 6.245)	0.0000	0.0000
block_typeLEADER-FOLLOWER	1.279	(-2.172, 4.776)	0.4573	0.5145
scale(PhysiologicalSynchrony_100)	-0.392	(-1.129, 0.345)	0.3062	0.3937
scale(Social_anxiety)	-1.981	(-4.001, 0.062)	0.0565	0.1017
scale(Perspective_taking)	-1.532	(-3.643, 0.604)	0.1554	0.2331
scale(Grasp_Time_Difference)	-3.113	(-3.937, -2.298)	0.0001	0.0003
scale(HRV_change)	0.092	(-1.036, 1.219)	0.8734	0.8734

5- Supplementary Results

Grasping asynchrony - The model included Block Type , Movement, BlockNovelty, StartTimeDifference, MT_variability, Social Anxiety, Perspective_Taking and HRV. The dependent variable GraspTimeDifference was log-transformed to obtain normally distributed values. Since the predictors StartTimeDifference and MT_variability were significantly correlated (r = 0.36, p = .030), to avoid multicollinearity issues, we ran a separate model including StartTimeDifference. In both models, none of the absolute difference predictors were significant, therefore were dropped in the trimmed models.

Model S1

$$Grasp\_Time\_Difference_{ij} = \beta_0 + \beta_1 \cdot Movement_i + \beta_2 \cdot BlockNovelty_i + \beta_3 \cdot Block\ Type_i + \beta_4 \cdot SUM\_HRV\_change_i + \beta_5 \cdot SUM\_Social\ Anxiety_i + \beta_6 \cdot SUM\_Perspective\_taking_i + \beta_7 \cdot SUM\_MovTimeVariability_i + b_{0j} + b_{1j} \cdot Block\ Type_i + b_{2j} \cdot Movement_i + \epsilon_{ij}$$

Model S1 ($R^2_{conditional}$ = 0.375, $R^2_{marginal}$ = 0.114, AIC = -782.98) showed a significantly better fit compared to the null model ($R^2_{conditional}$ = 0.237, $R^2_{marginal}$ = 0.00, AIC = -730.30, X^2 =76.67, df = 12, p < .0001). The model revealed a significant effect of Block Type (b = 0.046, CI [0.016,

0.076], p = . 008), indicating that GraspTimeDifference was smaller in the Free blocks than in the Leader-Follower ones, and a significant positive effect of MT_variability (b = 0.036, CI [0.024, 0.047], p < .001), see Figure 4a and Table 1. The model including StartTimeDifference failed to reveal any significant effect (see Supplementary Materials). Overall, this analysis revealed that participants were less able to touch the bottles at the same time when one of them was designed as the leader and the other had to follow compared to when they were free to adapt to each other. Moreover, participants were better at synchronizing when they reduced their movement time variability, thus making themselves more predictable.

Start asynchrony - This analysis estimates the impact of Block Type , Movement, BlockNovelty, Social Anxiety, Perspective_Taking, and HRV_change on the time difference between the participants movement start. The dependent variable StartTimeDifference was log-transformed to obtain normally distributed values. In the saturated model none of the absolute difference predictors were significant, therefore they were dropped in the trimmed model while maintaining their sum values.

Model S2

$$Start\_Time\_Difference_{ij} = \beta_0 + \beta_1 \cdot Movement_i + \beta_2 \cdot BlockNovelty_i + \beta_3 \cdot Block\ Type_i + \beta_4 \cdot SUM\_HRV\_change_i + \beta_5 \cdot SUM\_Social\ Anxiety_i + \beta_6 \cdot SUM\_Perspective\_taking_i + b_{0j} + b_{1j} \cdot Block\ Type_i + \epsilon_{ij}$$

Model S2 ($R^2_{conditional}$ = 0.63, $R^2_{marginal}$ = 0.22, AIC = -646.63) significantly outperformed the null model ($R^2_{conditional}$ = 0.34, $R^2_{marginal}$ = 0.00, AIC = -396.53, X^2 =266.1, df = 8, p < .0001). The model showed significant effects of Movement (b = -0.030, CI [-0.051, -0.01], p = 0.005) and Block Type (b = 0.153, CI [0.105, 0.20], p < 0.001). StartTimeDifference was lower in Same compared to Opposite blocks and in Free compared to Leader-Followerblocks, see Figure 4b and Table 1. The effect of Movement suggests that complementary actions require more motor preparation time than imitative actions, likely due to a conflict between competing motor representations. The effect of Block Type indicates that, as anticipated, in Leader-Follower blocks, the follower needed to wait for the leader to initiate the action in order to understand the goal. In addition, StartTimeDifference was positively related to Social Anxiety (b = 0.043, CI [0.008, 0.077], p = 0.033), indicating that socially anxious dyads were less able to synchronize their starting movement. Finally, there was a tendency toward significance for a negative effect of Perspective_taking (b = -0.037, CI [-0.073, - 0.002], p = . 054), although it did not survive the fdr correction.

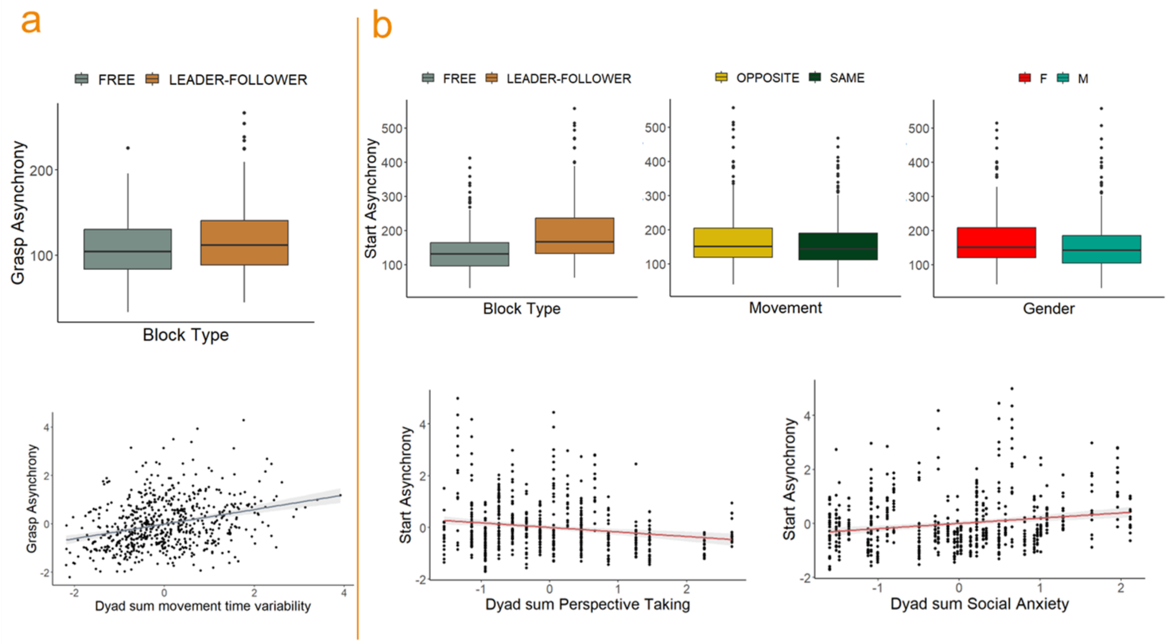

Figure S6 - Behavioural results. **Panel a**: Significant effects of Block Type (upper plot) and dyad level Movement Time Variability (lower plot) on Grasping Asynchrony. **Panel b**: Significant effects of Block Type, Movement, and Gender (upper plot) and dyad level Perspective Taking and Social Anxiety (lower plot) on Start Asynchrony.
